# MiR-150 blunts cardiac dysfunction in mice with cardiomyocyte loss of β_1_-adrenergic receptor/β-arrestin signaling and controls a unique transcriptome

**DOI:** 10.1038/s41420-022-01295-9

**Published:** 2022-12-30

**Authors:** Bruno Moukette, Satoshi Kawaguchi, Marisa N. Sepulveda, Taiki Hayasaka, Tatsuya Aonuma, Suthat Liangpunsakul, Lei Yang, Rohan Dharmakumar, Simon J. Conway, Il-man Kim

**Affiliations:** 1 Department of Anatomy, Cell Biology and Physiology, Indiana University School of Medicine, Indianapolis, IN USA; 2 Division of Gastroenterology and Hepatology, Indiana University School of Medicine, Indianapolis, IN USA; 3 Roudebush Veterans Administration Medical Center, Indianapolis, IN USA; 4 Herman B Wells Center for Pediatric Research, Indiana University School of Medicine, Indianapolis, IN USA; 5 Krannert Cardiovascular Research Center, Indiana University School of Medicine, Indianapolis, IN USA; 6 Present Address: Division of Cardiology, Nephrology, Pulmonology, and Neurology, Department of Internal Medicine, Asahikawa Medical University, Asahikawa, Hokkaido Japan

**Keywords:** Receptor pharmacology, Apoptosis, Extracellular signalling molecules, Heart failure, Non-coding RNAs

## Abstract

The β_1_-adrenergic receptor (β_1_AR) is found primarily in hearts (mainly in cardiomyocytes [CMs]) and β-arrestin-mediated β_1_AR signaling elicits cardioprotection through CM survival. We showed that microRNA-150 (miR-150) is upregulated by β-arrestin-mediated β_1_AR signaling and that CM miR-150 inhibits maladaptive remodeling post-myocardial infarction. Here, we investigate whether miR-150 rescues cardiac dysfunction in mice bearing CM-specific abrogation of β-arrestin-mediated β_1_AR signaling. Using CM-specific transgenic (TG) mice expressing a mutant β_1_AR (G protein-coupled receptor kinase [GRK]^–^β_1_AR that exhibits impairment in β-arrestin-mediated β_1_AR signaling), we first generate a novel double TG mouse line overexpressing miR-150. We demonstrate that miR-150 is sufficient to improve cardiac dysfunction in CM-specific GRK^–^β_1_AR TG mice following chronic catecholamine stimulation. Our genome-wide circular RNA, long noncoding RNA (lncRNA), and mRNA profiling analyses unveil a subset of cardiac ncRNAs and genes as heretofore unrecognized mechanisms for beneficial actions of β_1_AR/β-arrestin signaling or miR-150. We further show that lncRNA Gm41664 and GDAP1L1 are direct novel upstream and downstream regulators of miR-150. Lastly, CM protective actions of miR-150 are attributed to repressing pro-apoptotic GDAP1L1 and are mitigated by pro-apoptotic Gm41664. Our findings support the idea that miR-150 contributes significantly to β_1_AR/β-arrestin-mediated cardioprotection by regulating unique ncRNA and gene signatures in CMs.

## Introduction

Chronic stimulation of β-adrenergic receptors (βARs) induces adverse cardiac remodeling via Gαs protein-dependent pathways such as protein kinase A (PKA) signaling [1–5], which is a therapeutic basis for βAR antagonists (β-blockers) in heart failure (HF) [6–8]. However, β-blockers can have detrimental effects [9, 10], and the actions of individual β-blockers are divergent [11]. Thus, a better understanding of signaling events downstream of βAR is needed for developing novel HF therapeutics. Evidence of one such signaling pathway was revealed in a study, which used wild-type (WT) littermates and three lines of cardiomyocyte (CM)-specific transgenic (TG) mice for β_1_AR. By generating CM-specific TG mouse models for WT β_1_AR, one β_1_AR mutant lacking PKA phosphorylation sites and Gαs coupling (PKA^**–**^β_1_AR; β-arrestin-biased β_1_AR), and the other β_1_AR mutant lacking G protein-coupled receptor kinase [GRK] phosphorylation sites and β-arrestin coupling (GRK^**–**^β_1_AR; Gαs-biased β_1_AR), this study showed that GRK^**–**^β_1_AR TG had greater cardiac apoptosis and more severe HF than WT and two other lines of β_1_AR TG mice following chronic catecholamine treatment. This previous report demonstrated that β-arrestin-mediated β_1_AR signaling (β_1_AR/β-arrestin signaling) has a major influence on cardiac function in HF [12]. We then showed that the nonselective β-blocker carvedilol (Carv) elicits protective effects via β_1_AR/β-arrestin signaling in the absence of Gαs protein activation [13]. We also identified miR-150 as activated by Carv-mediated β_1_AR/β-arrestin protective signaling [14]. Distinct from β_2_AR expression, β_1_AR is found primarily in hearts (mainly in CMs), and β_1_AR/β-arrestin signaling confers protective effects in part due to increased CM survival [12]. However, despite the importance of the βAR/β-arrestin pathway in HF and our discovery of biased ligands to selectively activate this pathway, the detailed molecular mechanisms by which β_1_AR/β-arrestin signaling confers cardiac protection and CM survival are poorly understood. This lack of mechanistic insight represents a critical barrier to exploiting β_1_AR/β-arrestin signaling for the treatment of HF.

Small noncoding RNAs (ncRNAs) [microRNAs; miRNAs or miRs) downregulate target mRNAs. Modulation of cardiac miR activity is thought to be a critical underlying mechanism of HF [15–19]. Stimulatingly, novel miR therapies are being investigated in clinical trials for other diseases [20–23] and HF [24]. Using a miR-150 knockout (KO) mouse model, we reported that β_1_AR/β-arrestin-responsive miR-150 is protective in myocardial infarction (MI) in part via suppressing CM apoptosis [25]. More recently, we demonstrated that CM-specific miR-150 conditional KO (cKO) mice augment apoptosis and maladaptive post-MI remodeling partially by inducing apoptotic genes in CMs [26]. Interestingly, miR-150 is downregulated in patients with many cardiovascular diseases (CVDs) [27–30] and diverse rodent models of HF [25, 31, 32]. MiR-150 is conserved between rodents and humans, and it is significantly associated with the severity and outcome in patients with HF [33]. Collectively, these previous studies support the clinical relevance and potential therapeutic application of miR-150 in HF. However, detailed mechanisms by which miR-150 is controlled and regulates HF remain elusive.

Long ncRNAs (lncRNAs) have emerged as crucial regulators of human diseases [34, 35]. Pharmaceutical companies have accordingly tried to develop lncRNA therapeutics [36–39]. Interestingly, lncRNAs function as endogenous competing endogenous RNAs (ceRNAs) that sponge miRs, thus activating target mRNAs of miRs [40]. We recently showed that Carv/β_1_AR/β-arrestin-responsive miR-150 overexpression attenuates maladaptive post-MI remodeling caused by a lncRNA MIAT [41], indicating that MIAT is a functionally important upstream negative regulator of cardiac miR-150. Gm41664 is a natural anti-sense lncRNA of *kinesin family member 5B* (*Kif5b*) and there has been no study on this anti-sense lncRNA. Directly related to this study, evidence establishing the functional link between Gm41664 and miR-150 in hearts and CMs is also lacking, and the upstream mechanisms modulating Gm41664 have not been defined.

Ganglioside Induced Differentiation Associated Protein 1 Like 1 (GDAP1L1) is a regulator of mitochondrial fission. Mitochondrial fission is required for maintaining mitochondrial functions including apoptosis [42]. GDAP1L1 is mainly expressed in the central nervous system [43]. Diseases associated with GDAP1L1 include Spinocerebellar Ataxia 36 and Charcot-Marie-Tooth Disease, and a known paralog of this gene is GDAP1 [44]. GDAP1L1 regulates cytokine production by activating mitogen-activated protein kinase (MAPK) and nuclear factor (NF)-κB pathways. GDAP1L1 mediates macrophage activation, thus promoting neutrophil chemotaxis and keratinocyte hyperproliferation [45]. However, little is known about the regulatory mechanisms of GDAP1L1 by ncRNAs. In particular, whether GDAP1L1 is functionally regulated by the β_1_AR/β-arrestin/Gm41664/miR-150 axis in HF and CM apoptosis remains unknown.

Using a novel double TG (DTG) mouse model, unbiased genome-wide ncRNA and gene profiling analyses, and CM studies, we demonstrate in the current study that (i) miR-150 overexpression in mice with CM-specific deficiency in β_1_AR/β-arrestin protective signaling mitigates cardiac dysfunction after catecholamine stimulation; (ii) the lncRNA, Gm41664 is a novel negative regulator of miR-150 and *Gdap1l1* is an unrecognized target of the Gm41664/miR-150 axis; (iii) the expression of Gm41664 or *Gdap1l1* is upregulated in CMs subjected simulated ischemia/reperfusion (hypoxia/reoxygenation) [sI/R (H/R)], concurrent with miR-150 downregulation; and (iv) the protective effects of miR-150 in CMs are mediated by the direct and functional inhibition of apoptotic *Gdap1l1*, and the apoptotic action of Gm41664 in CMs is alleviated by the direct and functional target, miR-150. Thus, the Gm41664/miR-150/*Gdap1l1* axis may be considered a novel therapeutic modality for HF associated with CM dysfunction.

## Results

### MiR-150 overexpression attenuates deterioration of cardiac function after chronic catecholamine stimulation in mice with CM-specific deficiency of β-arrestin-mediated β_1_AR signaling

**Fig. 1:**
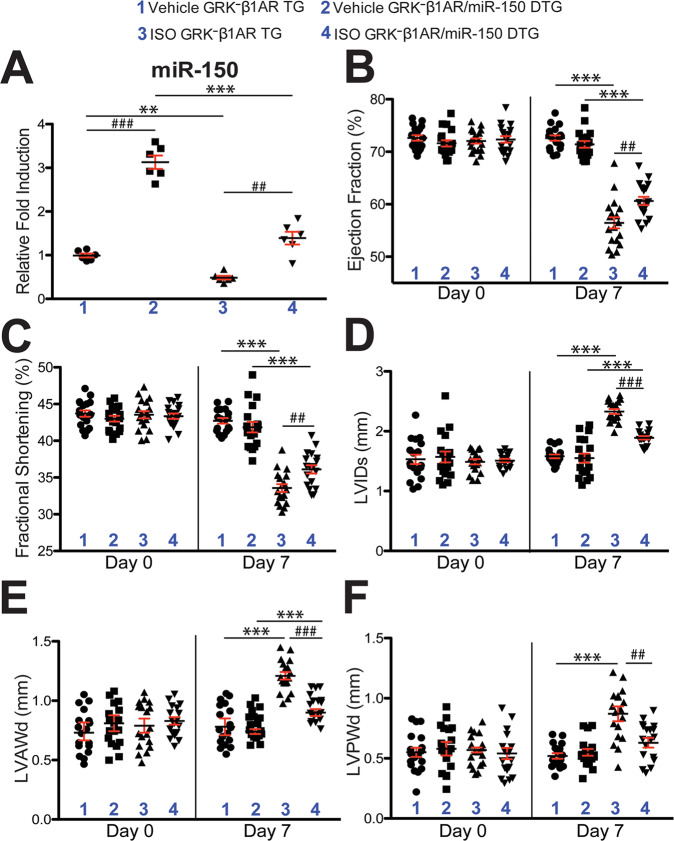
Cardiomyocyte-specific overexpression of GRK^−^β_1_AR exacerbates cardiac dysfunction following chronic isoproterenol treatment, which is in part rescued by miR-150. **A** MiR-150 expression in left ventricles (LVs) from cardiomyocyte (CM)-specific GRK^−^β_1_AR transgenic (TG) and GRK^−^β_1_AR;miR-150 double TG (DTG) mice at 1 week after vehicle or isoproterenol (ISO) infusion. *N* = 6. Data are presented as fold induction of miR-150 expression normalized to U6 snRNA. Two-way ANOVA with Tukey multiple comparison test. **P < 0.01 or ***P < 0.001 vs. vehicle; ^##^*P* < 0.01 or ^###^*P* < 0.001 vs. GRK^−^β_1_AR TG. **B**–**F** Transthoracic echocardiography was performed in 4 experimental groups (vehicle and ISO of GRK^−^β_1_AR TG and GRK^−^β_1_AR;miR-150 DTG) at week 0 and 1 post-treatment. Quantification of LV ejection fraction (**B**), fractional shortening (**C**), internal diameter, systole (LVIDs: **D**), anterior wall thickness, diastole (LVAWd: **E**), and posterior wall thickness, diastole (LVPWd: **F**) is presented. *N* = 18 per group. Two-way repeated-measures ANOVA with Bonferroni post hoc test. ***P < 0.001 vs. vehicle; ^##^*P* < 0.01 or ^###^*P* < 0.001 vs. GRK^−^β_1_AR TG. All data are presented as mean ± SEM.

HF is a condition associated with excessive sympathetic stimulation. A previous study showed that β-arrestin-mediated β_1_AR signaling elicits cardioprotection by generating a CM-specific TG mouse model overexpressing a mutant β_1_AR that lacks phosphorylation sites on β_1_AR by GRKs and demonstrating significant deterioration in cardiac function after chronic catecholamine stimulation [12]. We previously reported that the CM-specific TG mice [hereinafter referred to as GRK^**–**^β_1_AR TG mice], which exhibit impaired β-arrestin-mediated β_1_AR protective signaling [12], failed to upregulate miR-150 in response to β-arrestin-biased β_1_AR ligands [14]. To test whether a β_1_AR/β-arrestin-responsive miR, miR-150 would confer protection against chronic isoproterenol (ISO) treatment in GRK^**–**^β_1_AR TG mice, we bred GRK^**–**^β_1_AR TG mice with miR-150 TG mice to generate a novel GRK^**–**^β_1_AR/miR-150 DTG mouse line. First, we confirm miR-150 overexpression by showing that the level of miR-150 is increased by ~3 folds in GRK^**–**^β_1_AR/miR-150 DTG mice compared to GRK^**–**^β_1_AR TG controls. We also observe miR-150 downregulation in the hearts of both GRK^**–**^β_1_AR/miR-150 DTG and GRK^**–**^β_1_AR TG mice subjected to 1 week of ISO (Fig. 1A). We further show that GRK^**–**^β_1_AR/miR-150 DTG mice have a normal cardiac function at baseline (Fig. 1B–F and Table S1). However, a significant enhancement in cardiac function is observed at 7 days after ISO treatment in GRK^**–**^β_1_AR/miR-150 DTG mice compared to GRK^**–**^β_1_AR TG mice. This functional improvement is indicated by increased ejection fraction (EF), fractional shortening (FS), cardiac output (CO), and stroke volume (SV), as well as reduced diastolic left ventricular interior diameter (LVIDd), systolic LVID (LVIDs), diastolic LV anterior wall thickness (LVAWd), systolic LVAW (LVAWs), diastolic LV posterior wall thickness (LVPWd), systolic LVPW (LVPWs), end-diastolic volume (EDV), and end-systolic volume (ESV) (Fig. 1B–F and Table S2). Lastly, our morphometric data reveal that GRK^**–**^β_1_AR/miR-150 DTG mice display significantly decreased heart weight (HW)/body weight (BW) and LV weight (LVW)/BW after chronic ISO treatment (Table S2) when compared with GRK^**–**^β_1_AR TG mice. Collectively, these findings indicate that genetic overexpression of miR-150 in GRK^**–**^β_1_AR TG mice relieves cardiac dysfunction under conditions of catecholamine excess, thereby establishing for the first time the direct in vivo functional interaction between β-arrestin-mediated β_1_AR protective signaling and miR-150 in the heart.

### MiR-150 overexpression in mice with CM-specific ablation of β-arrestin-mediated β_1_AR signaling largely corrects replacement fibrosis and cell death after chronic catecholamine treatment

**Fig. 2:**
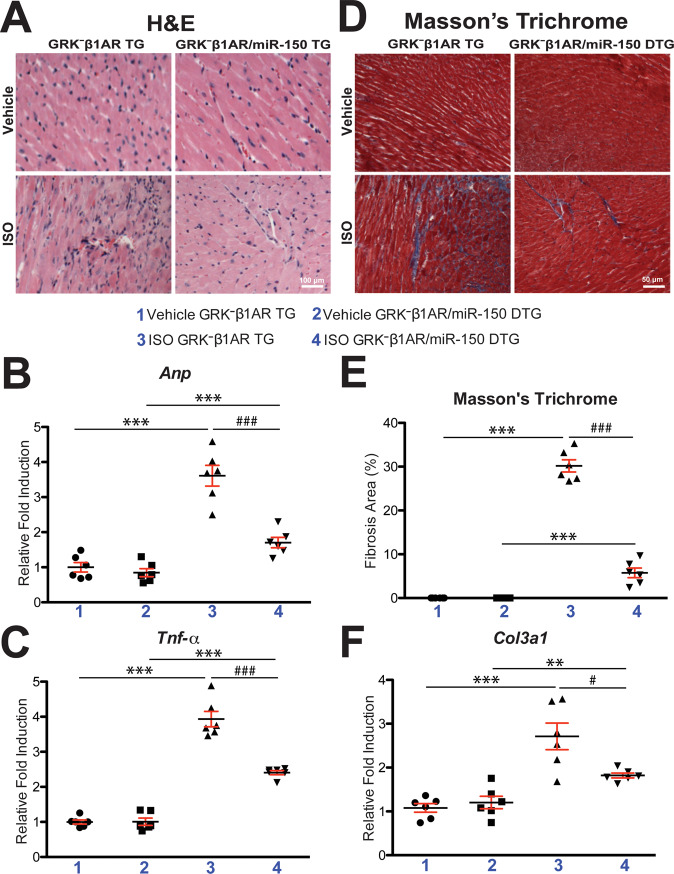
Cardiomyocyte-specific overexpression of GRK^−^β_1_AR induces cardiac stress and fibrosis after chronic isoproterenol treatment, which is alleviated by miR-150. **A** Representative hematoxylin and eosin (H&E) images from heart sections at 1-week post-treatment show a decrease in abnormal architecture and cellular integrity, and in disorganized structure in ISO GRK^−^β_1_AR;miR-150 DTG hearts compared to ISO GRK^−^β_1_AR TG controls- Scale bar: 100 μm. **B**, **C** Real-Time Quantitative Reverse Transcription (QRT)-PCR analyses of *Anp* (**B**) and *Tnf-α* (**C**) expression for cardiac damage and inflammation in GRK^−^β_1_AR;miR-150 DTG hearts compared to GRK^−^β_1_AR TG controls at 1-week post-treatment. *N* = 6 per group. Data are presented as fold induction of gene expression normalized to glyceraldehyde-3-phosphate dehydrogenase (*Gapdh*). Two-way ANOVA with Tukey multiple comparison test. ***P < 0.001 vs. vehicle; ^###^*P* < 0.001 vs. ISO GRK^−^β_1_AR TG. **D**, **E** Representative Masson’s trichrome images in heart sections from 4 experimental groups at 1-week post-treatment (**D**) and fibrosis quantification in whole left ventricles (LVs) (**E**). Scale bar: 50 μm. *N* = 6 per group. Two-way ANOVA with Tukey multiple comparison test. ***P < 0.001 vs. vehicle; ^###^*P* < 0.001 vs. ISO GRK^−^β_1_AR TG. **F** QRT-PCR analysis of fibrotic *Col3a1* expression in GRK^−^β_1_AR;miR-150 DTG hearts compared to GRK^−^β_1_AR TG controls at 1-week post-treatment. *N* = 6 per group. Data are presented as fold induction of gene expression normalized to *Gapdh*. Two-way ANOVA with Tukey multiple comparison test. **P < 0.01 or ***P < 0.001 vs. vehicle; ^#^*P* < 0.05 vs. ISO GRK^−^β_1_AR TG. All data are presented as mean ± SEM.

**Fig. 3:**
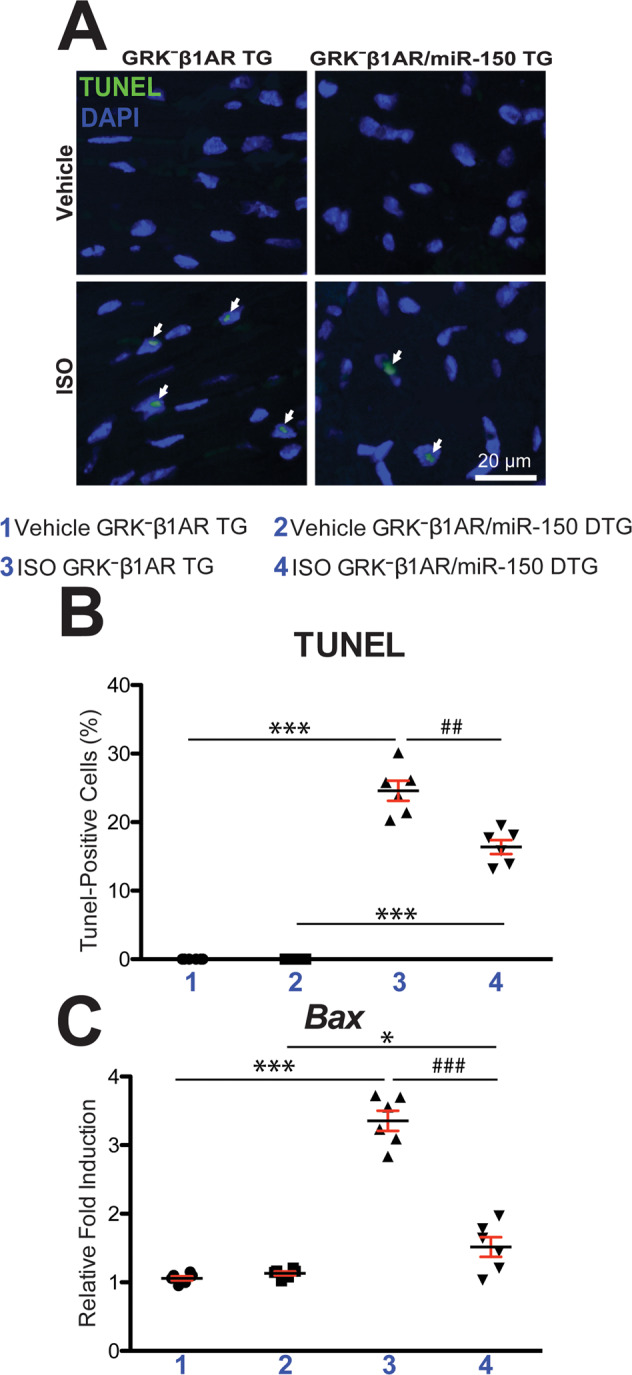
Cardiomyocyte-specific overexpression of GRK^−^β_1_AR augments cardiac apoptosis after chronic isoproterenol treatment, which is repressed by miR-150. **A**, **B** Representative terminal deoxynucleotidyl transferase dUTP nick end labeling (TUNEL) staining images in heart sections at 1-week post-treatment (**A**) and apoptosis quantification in six 40× fields (**B**). Scale bar: 20 μm. **C** QRT-PCR analysis of pro-apoptotic *Bax* expression in GRK^−^β_1_AR;miR-150 DTG hearts compared to GRK^−^β_1_AR TG controls at 1-week post-treatment. *N* = 6 per group. Data are presented as fold induction of gene expression normalized to *Gapdh*. Two-way ANOVA with Tukey multiple comparison test. *P < 0.05 or ***P < 0.001 vs. vehicle; ^##^*P* < 0.01 or ^###^*P* < 0.001 vs. ISO GRK^−^β_1_AR TG. All data are presented as mean ± SEM.

To determine if miR-150 partially rescues cardiac deterioration in GRK^–^β_1_AR TG mice after chronic catecholamine stimulation, we examined cardiac damage, fibrosis, and cell death in GRK^**–**^β_1_AR/miR-150 DTG mice. We find that GRK^**–**^β_1_AR/miR-150 DTG hearts significantly reduce the loss of normal architecture and cellular integrity, as well as necrosis and infiltration of additional nuclei after 1 week of ISO stimulation compared to GRK^**–**^β_1_AR TG hearts (Fig. 2A). GRK^**–**^β_1_AR/miR-150 DTG hearts also have a significant decrease in mRNA levels of fetal *Anp* (Fig. 2B) after 1 week of ISO stimulation compared to GRK^**–**^β_1_AR TG hearts. The expression of inflammatory *Tnf-α* is also decreased in GRK^**–**^β_1_AR/miR-150 DTG hearts (Fig. 2C) compared to GRK^**–**^β_1_AR hearts. By performing Masson’s trichrome staining of the hearts, we observe significantly reduced interstitial fibrosis post-ISO treatment in GRK^**–**^β_1_AR/miR-150 DTG hearts than GRK^**–**^β_1_AR TG hearts (Fig. 2D, E), consistent with decreased expression of fibrotic *Col3a1* in GRK^**–**^β_1_AR/miR-150 DTG hearts compared to GRK^**–**^β_1_AR TG controls (Fig. 2F). Lastly, GRK^**–**^β_1_AR/miR-150 DTG hearts contain significantly decreased numbers of TUNEL-positive cells post-ISO treatment (Fig. 3A, B) and a significant decrease in mRNA levels of apoptotic *Bax* (Fig. 3C) compared to GRK^**–**^β_1_AR TG hearts. Altogether, our data indicate that miR-150 attenuates cardiac dysfunction caused by abrogation of β-arrestin-mediated β_1_AR signaling and that miR-150 represents a crucial downstream mechanism by which β_1_AR-mediated β-arrestin signaling confers beneficial remodeling in failing hearts by inhibiting CM cell death.

### CM-specific abrogation in β-arrestin-mediated β_1_AR signaling decreases cardiac circRNA-31100 and circRNA-32197 after chronic catecholamine stimulation, which is reversed by miR-150

Although miR-150 is involved in human HF [33], the mechanistic details of its actions are largely unknown. To investigate how cardiac miR-150 exerts its function in mice with CM-specific deficiency in β-arrestin-mediated β_1_AR signaling, we performed circular noncoding RNA (circRNA) profiling of left ventricles (LVs) from GRK^**–**^β_1_AR TG and GRK^**–**^β_1_AR/miR-150 DTG mice subjected to the vehicle or ISO treatment for 1 week. We provide further details in Supplementary Results to describe Fig. S1, Fig. S2, and Table S3. To identify novel circRNAs that regulate miR-150’s protective actions in mice with the selective abrogation of β-arrestin-mediated β_1_AR signaling in CMs, we filtered 4 dysregulated circRNAs from our array data based on the correlation between cardiac phenotypes (Figs. 1–3) and circRNA signatures from ISO GRK^**–**^β_1_AR TG vs. vehicle GRK^**–**^β_1_AR TG (Fig. S3A, B, I dataset) or ISO GRK^**–**^β_1_AR/miR-150 DTG vs. ISO GRK^**–**^β_1_AR TG (Fig. S3A, B, II dataset). The rationale to focus on the 2 potentially deleterious circRNAs is that they are upregulated in ISO GRK^**–**^β_1_AR TG vs. Vehicle GRK^**–**^β_1_AR TG (i.e., phenotypic effects of CM-specific deficiency in β_1_AR/β-arrestin signaling), but downregulated in ISO GRK^**–**^β_1_AR/miR-150 DTG vs. ISO GRK^**–**^β_1_AR TG (i.e., protective effects of miR-150 overexpression) (Fig. S3A, B). The rationale to focus on the other 2 potentially beneficial circRNAs (*circRNA-31100* and *circRNA-32197*) is that they are downregulated in ISO GRK^**–**^β_1_AR TG vs. vehicle GRK^**–**^β_1_AR TG (i.e., phenotypic effects of CM-specific deficiency in β_1_AR/β-arrestin signaling), but upregulated in ISO GRK^**–**^β_1_AR/miR-150 DTG vs. ISO GRK^**–**^β_1_AR TG (i.e., protective effects of miR-150 overexpression) (Fig. S3A, B). Using QRT-PCR analyses for the 4 circRNAs, we validate that LV *circRNA-31100* and *circRNA-32197* are upregulated in ISO GRK^**–**^β_1_AR/miR-150 DTG mice compared to ISO GRK^**–**^β_1_AR TG controls (Fig. S3C–E). Notably, we demonstrate that the expression of *circRNA-31100* and *circRNA-32197* is downregulated in GRK^**–**^β_1_AR TG hearts post-ISO treatment (Fig. S3D, E), consistent with cardiac miR-150 downregulation (Fig. 1A). Thus, our circRNA profiling and validation analyses suggest that *circRNA-31100* and *circRNA-32197* are novel regulators that mediate miR-150’s protective actions in mice with CM-specific deficiency in β-arrestin-mediated β_1_AR signaling after chronic ISO treatment.

We next find that *circRNA-31100* is an exonic circRNA of *Epas1* and has a putative binding site for miR-547-5p, miR-152-3p, miR-7059-3p, miR-6539, and miR-152-5p (Table S3; see Down_GRK^**–**^β_1_AR ISO vs Vehicle sheet and Up_ISO DTG vs GRK sheet). We also observe that *circRNA-32197* is a sense-overlapping circRNA of lncRNA *Malat1* and has a putative binding site for miR-495-3p, miR-505-3p, miR-1954, miR-6903-5p, and miR-3092-5p (Table S3; see Down_GRK^**–**^β_1_AR ISO vs Vehicle sheet and Up_ISO DTG vs GRK sheet). Interestingly, our prediction analyses fail to find a putative binding site for miR-150 in *circRNA-31100* and *circRNA-32197*, indicating that these circRNAs function in a miR-150-independent manner.

### CM-specific ablation in β-arrestin-mediated β_1_AR signaling activates cardiac lncRNAs (Gm41664, Gmds, and Tspan2os) after chronic catecholamine stimulation, which is reversed by miR-150 overexpression

**Fig. 4:**
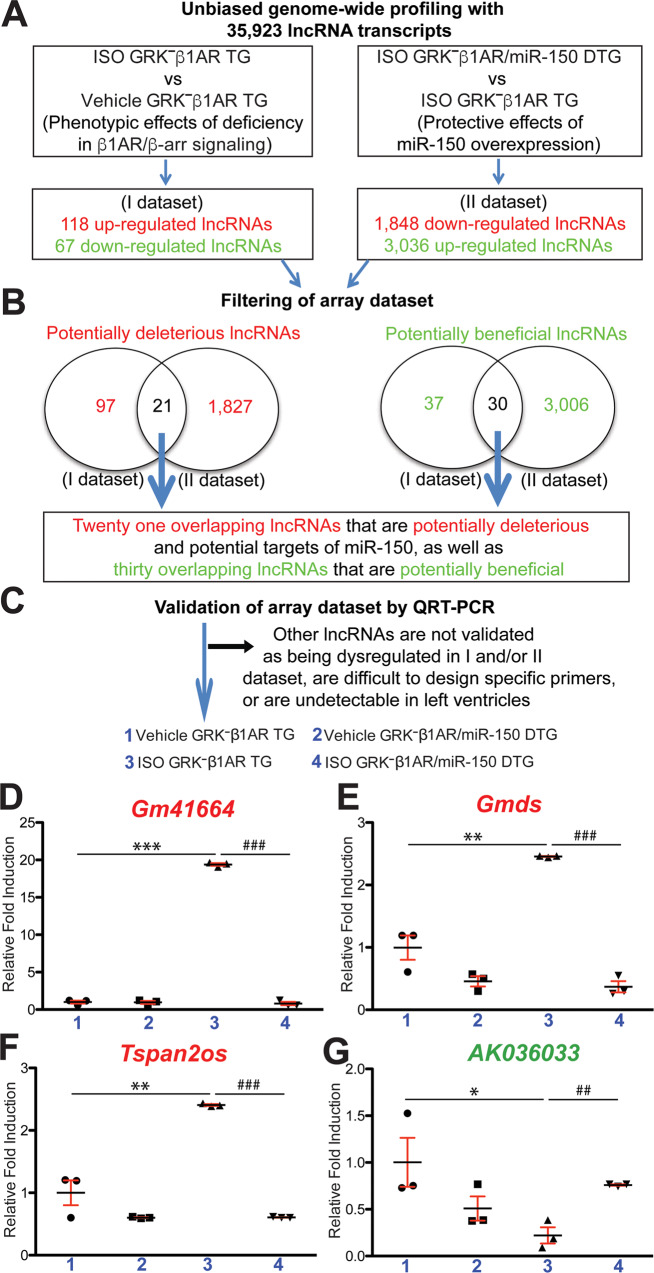
Genome-wide long noncoding RNA profiling in GRK^−^β_1_AR TG and GRK^−^β_1_AR;miR-150 DTG mice identifies novel long noncoding RNAs that are controlled by β_1_AR/β-arrestin-mediated signaling and regulate miR-150. **A**, **B** Genome-wide profiling and filtering strategies of array dataset based on the correlation between transcript signatures and cardiac phenotypes. Twenty-one dysregulated (DE) long noncoding RNAs (lncRNAs), which are upregulated in the I dataset (ISO GRK^−^β_1_AR TG compared to vehicle GRK^−^β_1_AR TG controls) but are downregulated in the II dataset (ISO GRK^−^β_1_AR;miR-150 DTG compared to ISO GRK^−^β_1_AR TG) at 1-week post-treatment, were chosen for further analyses. Thirty other DE lncRNAs, which are downregulated in the I dataset (ISO GRK^−^β_1_AR TG compared to vehicle GRK^−^β_1_AR TG controls) but are upregulated in the II dataset (ISO GRK^−^β_1_AR;miR-150 DTG compared to ISO GRK^−^β_1_AR TG) at 1-week post-treatment, were chosen for further analyses. *N* = 3 per group. **C**–**G** Validation strategy of array dataset. Three potentially deleterious DE lncRNAs (Gm41664, Gmds, and Tspan2os) were validated by QRT-PCR analyses in LVs from GRK^−^β_1_AR TG and GRK^−^β_1_AR;miR-150 DTG mice at 1-week post-treatment (**D**–**F**). The other potentially beneficial DE lncRNA (AK036033) was validated by QRT-PCR analyses in LVs from GRK^−^β_1_AR TG and GRK^−^β_1_AR;miR-150 DTG mice at 1-week post-treatment (**G**). Of note, other lncRNAs are not validated as being dysregulated as presented in (**B**), are difficult to design specific primers, or are undetectable in LVs. Data are shown as fold induction of lncRNA expression normalized to *Gapdh*. *N* = 3 per group. Two-way ANOVA with Tukey multiple comparison test. *P < 0.05, **P < 0.01, or ***P < 0.001 vs. vehicle; ^##^*P* < 0.01 or ^###^*P* < 0.001 vs. ISO GRK^−β^_1_AR TG. All data are presented as mean ± SEM.

Because we failed to identify circRNAs that directly regulate miR-150, we hypothesized that lncRNAs may directly regulate miR-150’s protective actions in mice with selective deficiency of β-arrestin-mediated β_1_AR signaling in CMs. We tested this hypothesis by conducting lncRNA microarray profiling in mouse LVs to unveil lncRNA signatures modulated by miR-150 overexpression in mice with CM-specific loss of β-arrestin-mediated β_1_AR signaling after chronic ISO stimulation. We provide further details in Supplementary Results to describe Fig. S4 and Table S4. To discover novel lncRNAs that control miR-150’s protective actions in mice with CM-specific deficiency in β-arrestin-mediated β_1_AR signaling, we filtered 51 dysregulated lncRNAs from our array data based on the correlation between cardiac phenotypes (Figs. 1–3) and lncRNA signatures from ISO GRK^**–**^β_1_AR TG vs. vehicle GRK^**–**^β_1_AR TG (Fig. 4A, B, I dataset) or ISO GRK^**–**^β_1_AR/miR-150 DTG vs. ISO GRK^**–**^β_1_AR TG (Fig. 4A, B, II dataset). The rationale to focus on the 21 potentially deleterious lncRNAs is that they are upregulated in ISO GRK^**–**^β_1_AR TG vs. vehicle GRK^**–**^β_1_AR TG (i.e., phenotypic effects of CM-specific deficiency in β_1_AR/β-arrestin signaling), but downregulated in ISO GRK^**–**^β_1_AR/miR-150 DTG vs. ISO GRK^**–**^β_1_AR TG (i.e., protective effects of miR-150 overexpression) (Fig. 4A, B). The rationale to focus on the other 30 potentially beneficial lncRNAs is that they are downregulated in ISO GRK^**–**^β_1_AR TG vs. vehicle GRK^**–**^β_1_AR TG (i.e., phenotypic effects of CM-specific deficiency in β_1_AR/β-arrestin signaling), but upregulated in ISO GRK^**–**^β_1_AR/miR-150 DTG vs. ISO GRK^**–**^β_1_AR TG (i.e., protective effects of miR-150 overexpression) (Fig. 4A, B). Using real-time QRT-PCR analyses, we validate among the 21 potentially deleterious lncRNAs that LV *Gm41664, Gmds*, and *Tspan2os* are downregulated in ISO GRK^**–**^β_1_AR/miR-150 DTG mice compared to ISO GRK^**–**^β_1_AR TG controls (Fig. 4C–F). Notably, we demonstrate that the expression of *Gm41664, Gmds*, and *Tspan2os* is upregulated in GRK^**–**^β_1_AR TG hearts post-ISO treatment (Fig. 4D–F), concurrent with cardiac miR-150 downregulation (Fig. 1A). Moreover, we validate among the 30 potentially beneficial lncRNAs that LV *AK036033* is upregulated in ISO GRK^**–**^β_1_AR/miR-150 DTG mice compared to ISO GRK^**–**^β_1_AR TG controls (Fig. 4C, G). Of note, we also show that *AK036033* expression is downregulated in GRK^**–**^β_1_AR TG hearts post-ISO treatment (Fig. 4G), consistent with cardiac miR-150 downregulation (Fig. 1A). Thus, our lncRNA profiling and validation analyses indicate that *Gm41664, Gmds, Tspan2os*, and *AK036033* are novel regulators that control miR-150’s protective actions in mice with CM-specific loss in β-arrestin-mediated β_1_AR signaling after chronic ISO treatment.

We next observe that *Gm41664* is a natural anti-sense lncRNA of *Kif5b* (Table S4; see Up_GRK^**–**^β_1_AR ISO vs Vehicle sheet and Down_ISO DTG vs GRK sheet). We used miR target prediction tools [46–49] and find many potential binding sites for miR-150 in *Gm41664*. We also find that *Gmds* is an exon-sense-overlapping lncRNA of *Gmds* gene locus (Table S4; see Up_GRK^**–**^β_1_AR ISO vs Vehicle sheet and Down_ISO DTG vs GRK sheet) and has no binding site for miR-150. Our further analyses reveal that *Tspan2os* is a natural anti-sense lncRNA of *Tspan2* (Table S4; see Up_GRK^**–**^β_1_AR ISO vs Vehicle sheet and Down_ISO DTG vs GRK sheet) and has no predicted binding site for miR-150. Lastly, we find that *AK036033* (also known as *9630028I04Rik*) is an intergenic lncRNA (Table S4; see Down_GRK^**–**^β_1_AR ISO vs Vehicle sheet and Up_ISO DTG vs GRK sheet) which has no miR-150 binding site. Taken together, our lncRNA profiling, validation, and miR-150 binding prediction analyses suggest that *Gm41664* is a novel direct regulator of miR-150 in HF associated with excess catecholamine stimulation.

### Opposing by CM-specific deficiency in β-arrestin-mediated β_1_AR signaling, miR-150 overexpression inhibits seven cardiac genes (Cspg5, Cfl1, Gdap1l1, Mfsd12, Arhgef39, Map2k7, and Cdk14), while activating cardiac Slitrk6 after chronic catecholamine stimulation

**Fig. 5:**
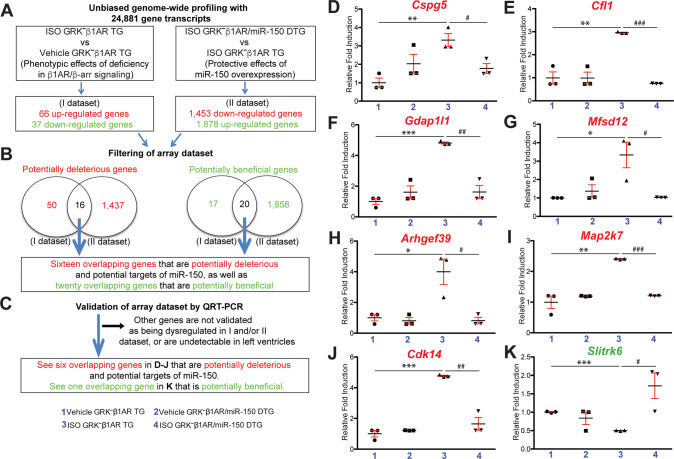
Genome-wide gene profiling in GRK^−^β_1_AR TG and GRK^−^β_1_AR;miR-150 DTG mice identifies novel genes that are regulated by β_1_AR/β-arrestin-mediated signaling and are miR-150’s targets. **A**, **B** Genome-wide profiling and filtering strategies of array dataset based on the correlation between transcript signatures and cardiac phenotypes. Sixteen dysregulated (DE) genes, which are upregulated in the I dataset (ISO GRK^−^β_1_AR TG compared to vehicle GRK^−^β_1_AR TG controls) but are downregulated in the II dataset (ISO GRK^−^β_1_AR;miR-150 DTG compared to ISO GRK^−^β_1_AR TG) at 1-week post-treatment, were chosen for additional analyses. Twenty other DE genes, which are downregulated in the I dataset (ISO GRK^−^β_1_AR TG compared to vehicle GRK^−^β_1_AR TG controls) but are upregulated in the II dataset (ISO GRK^−^β_1_AR;miR-150 DTG compared to ISO GRK^−^β_1_AR TG) at 1-week post-treatment, were chosen for further analyses. *N* = 3 per group. **C**–**K** Validation strategy of array dataset. Seven potentially deleterious DE genes (*Cspg5*, *Cfl1*, *Gdap1l1*, *Mfsd12*, *Arhgef39*, *Map2k7*, and *Cdk14*) were validated by QRT-PCR analyses in LVs from GRK^−^β_1_AR TG and GRK^−^β_1_AR;miR-150 DTG mice at 1-week post-treatment (**D–J**). The other potentially beneficial DE gene (*Slitrk6*) was validated by QRT-PCR analyses in LVs from GRK^−^β_1_AR TG and GRK^−^β_1_AR;miR-150 DTG mice at 1-week post-treatment (**K**). Of note, other genes are not validated as being dysregulated as shown in (**B**) or are undetectable in LVs. Data are presented as fold induction of gene expression normalized to *Gapdh*. *N* = 3 per group. Two-way ANOVA with Tukey multiple comparison test. *P < 0.05, **P < 0.01, or ***P < 0.001 vs. vehicle; ^#^*P* < 0.05, ^##^*P* < 0.01, or ^###^*P* < 0.001 vs^.^ ISO GRK^−^β_1_AR TG. All data are presented as mean ± SEM.

We next performed genome-wide mRNA profiling of mouse LVs to identify novel direct target genes of miR-150. LV tissues from GRK^**–**^β_1_AR TG and GRK^**–**^β_1_AR/miR-150 DTG mice subjected to the vehicle or ISO were investigated at 1-week post-treatment. We provide further details in Supplementary Results to describe Fig. S5, Fig. S6, Fig. S7, and Table S5. To define novel candidate targets that regulate miR-150’s protective actions in mice with CM-specific loss in β-arrestin-mediated β_1_AR signaling, we filtered 36 dysregulated genes from our array data based on the correlation between cardiac phenotypes (Figs. 1–3) and gene signatures from ISO GRK^**–**^β_1_AR TG vs. vehicle GRK^**–**^β_1_AR TG (Fig. 5A, B, I dataset) or ISO GRK^**–**^β_1_AR/miR-150 DTG vs. ISO GRK^**–**^β_1_AR TG (Fig. 5A, B, II dataset). The rationale to focus on the 16 potentially deleterious genes (Table S5: see red-shaded columns) is that they are upregulated in ISO GRK^**–**^β_1_AR TG vs. vehicle GRK^**–**^β_1_AR TG (i.e., phenotypic effects of CM-specific deficiency in β_1_AR/β-arrestin signaling), but downregulated in ISO GRK^**–**^β_1_AR/miR-150 DTG vs. ISO GRK^**–**^β_1_AR TG (i.e., protective effects of miR-150 overexpression) (Fig. 5A, B). The rationale to focus on the other 20 potentially beneficial genes (Table S5: see green-shaded columns) is that they are downregulated in ISO GRK^**–**^β_1_AR TG vs. vehicle GRK^**–**^β_1_AR TG (i.e., phenotypic effects of CM-specific deficiency in β_1_AR/β-arrestin signaling), but upregulated in ISO GRK^**–**^β_1_AR/miR-150 DTG vs. ISO GRK^**–**^β_1_AR TG (i.e., protective effects of miR-150 overexpression) (Fig. 5A, B).

Using QRT-PCR analyses for validating the expression of the 36 filtered genes, we observe that *Cspg5, Cfl1, Gdap1l1, Mfsd12, Arhgef39, Map2k7,* and *Cdk14* in LVs are downregulated in ISO GRK^**–**^β_1_AR/miR-150 DTG mice compared to ISO GRK^**–**^β_1_AR TG controls (Fig. 5C–J). However, these seven genes are upregulated in GRK^**–**^β_1_AR TG hearts post-ISO treatment (Fig. 5D–J), concurrent with cardiac miR-150 downregulation (Fig. 1A). Moreover, we validate among the 20 potentially beneficial genes that LV *Slitrk6* is upregulated in ISO GRK^**–**^β_1_AR/miR-150 DTG mice compared to ISO GRK^**–**^β_1_AR TG controls (Fig. 5C, K). Of note, we also show that *Slitrk6* expression is downregulated in GRK^**–**^β_1_AR TG hearts post-ISO treatment (Fig. 5K), consistent with cardiac miR-150 downregulation (Fig. 1A). Using bioinformatic miR target prediction tools [46–49], we detect multiple putative binding sites for miR-150 in the *Gdap1l1* locus, but not for other aforementioned dysregulated genes. Therefore, our gene profiling, validation, and miR-150 binding prediction analyses suggest that *Gdap1l1* is a novel downstream target that directly regulates miR-150’s protective actions in mice with CM-specific loss in β-arrestin-mediated β_1_AR signaling after chronic ISO stimulation.

### A lncRNA, Gm41664 is a novel direct upstream regulator of miR-150 and GDAP1L1 is a novel direct target of miR-150

**Fig. 6:**
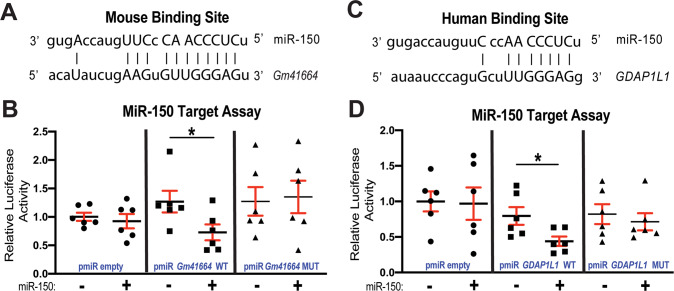
MiR-150 interacts with *Gm41664* and *GDAP1L1*. **A** Mouse *Gm41664* has the strongest miR-150 binding site with 12-mer complementary sequences. MiR-150 seed pairing in the target region and complementary sequences outside the seed region are presented as vertical lines. **B** MiR-150’s capability of directly repressing the activity of luciferase (LUC) reporter constructs that include either wild-type (WT) or mutated (MUT) binding sites for *Gm41664*. Transfection with or without miR-150 mimic in H9c2 cells is shown as + or −. The LUC activity of Firefly was normalized to the LUC activity of Renilla and compared with empty vector measurements. *N* = 6. Unpaired two-tailed *t*-test. **C** Human *GDAP1L1* has a conserved miR-150 binding site in the 3’-untranslated region (3’UTR). MiR-150 seed pairing in the target region and complementary sequences outside the seed region are presented as vertical lines. **D** The direct ability of miR-150 to inhibit the activity of LUC reporter constructs that include either WT or MUT binding sites for *GDAP1L1*. Transfection with or without miR-150 mimic in AC16 cells is indicated as + or −. The LUC activity of Firefly was normalized to the LUC activity of Renilla and compared to empty vector measurements. *N* = 6. Unpaired two-tailed *t*-test. *P < 0.05 vs. miR mimic control.

We next examined whether *Gm41664* interacts with miR-150. Our miR target prediction analysis reveals that mouse *Gm41664* has 33 putative binding sites for miR-150. Among all predicted binding sites, we identify the strongest binding site for miR-150 in *Gm41664* with 8-mer complementary sequences within the seed region of miR-150 and 4-mer complementary sequences outside the seed region (Fig. 6A). To test if *Gm41664* is a direct regulator of miR-150, we co-transfected miR-150 mimics and constitutively active luciferase (LUC) reporter constructs including the binding site of miR-150 in *Gm41664* (Fig. 6A). We find the repressed LUC activity by miR-150 for the WT *Gm41664* reporter. Upon mutating seed binding sites for miR-150, miR-150 does not affect LUC activity (Fig. 6B), suggesting that *Gm41664* and miR-150 bind in a sequence-specific manner.

We also find that mouse and human genes of GDAP1L1 contain 8 and 15 miR-150 binding sites, respectively, indicating the conserved regulation of *Gdap1l1* by miR-150 and their roles in mice and humans. Interestingly, we identify a potential conserved binding site for miR-150 in the human *GDAP1L1* 3’-untranslated region (UTR) (Fig. 6C). To examine whether *GDAP1L1* is a direct target of miR-150 inhibition, we transfected human CMs with constitutively active LUC reporter constructs including the conserved binding site of miR-150 in human *GDAP1L1* and miR-150 mimics. We find the repressed LUC activity by miR-150 for the WT *GDAP1L1* reporter. Upon mutating seed binding sites for miR-150, the LUC reporter is not sensitive to miR-150 overexpression (Fig. 6D), indicating that miR-150 directly suppresses *GDAP1L1* with the specific dependence of target sites. Therefore, our data suggest that *Gm41664* is a novel direct regulator of miR-150 and that *Gdap1l1* is a novel direct target of miR-150.

### Gm41664 negatively regulates cardiomyocyte survival partially by functionally inhibiting anti-apoptotic miR-150

Given that cardiac miR-150 is downregulated in CM-specific GRK^**–**^β_1_AR TG mice treated with ISO concurrent with Gm41664 upregulation (Fig. 1A and Fig. 4D) and that miR-150 is decreased in CMs subjected to low oxygen conditions [25], we next focused on CMs to test if the novel regulator of miR-150, Gm41664 is inversely regulated in CMs subjected to sI/R (H/R) conditions. Indeed, lncRNA Gm41664 is increased in mouse CMs after sI/R (H/R) (Fig. S8A), which is in agreement with our in vivo result post-ISO (Fig. 4D). Although Gm41664 and miR-150 interact in vitro (Fig. 6A, B) and their correlative relationship in CMs is shown, the functional relationship between these two ncRNAs has not been fully established. We first observe that CM miR-150 is upregulated after Gm41664 knockdown (Fig. S8B) while miR-150 knockdown does not significantly affect the expression of Gm41664 (*P* = 0.166). Our data suggest that Gm41664 suppresses miR-150 in CMs, presumably acting via a target RNA‐directed miR decay mechanism with multiple binding sites [50]. This idea is supported by the fact that Gm41664 includes 33 putative binding sites of miR-150 and that Gm41664 interacts with miR-150 in vitro (Fig. 6A, B).

**Fig. 7:**
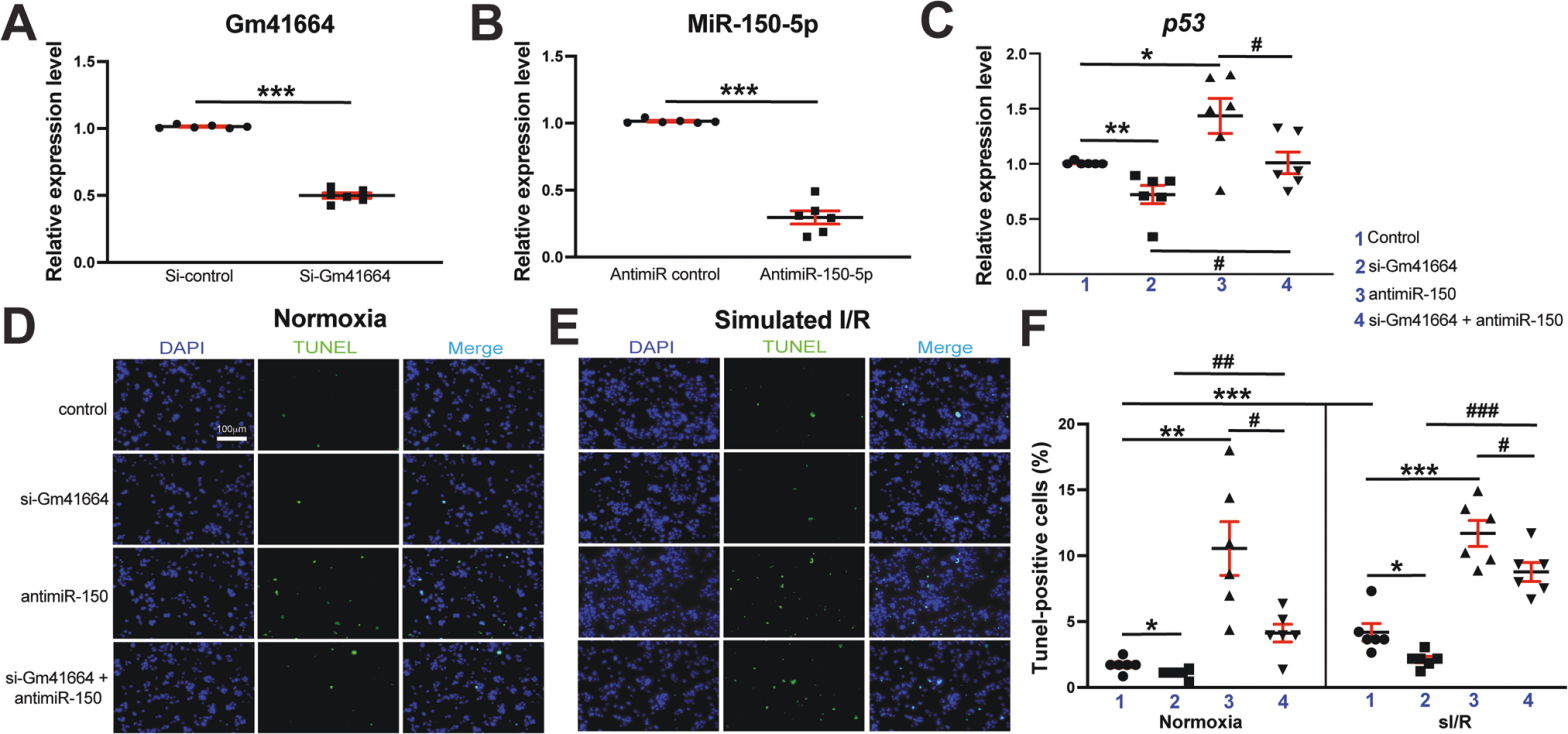
MiR-150 is required for Gm41664-dependent induction of cardiomyocyte apoptosis. **A**, **B** HL-1 cells were transfected with control scramble siRNA (si-control) or Gm41664 siRNA (si-Gm41664) (**A**) and with anti-miR control scramble or anti-miR-150 (**B**). QRT-PCR for Gm41664 (**A**) or miR-150 (**B**) was conducted to check the knockdown efficiency. Data were normalized to *Gapdh* (**A**) or *U6 snRNA* (**B**) and expressed relative to controls. *N* = 6 per group. Unpaired two-tailed *t*-test. ***P < 0.001 vs. si-control or anti-miR control. **C** QRT-PCR expression analysis of pro-apoptotic *p53* in cardiomyocytes (CMs) transfected with 4 different groups as indicated. *N* = 6. *p53* expression compared to *Gapdh* was calculated using 2^−ΔΔCt^, and data are presented as fold induction of *p53* expression levels normalized to control (si-control or anti-miR control). One-way ANOVA with Tukey multiple comparison test. *P < 0.05 or **P < 0.01 vs. control. ^#^*P* < 0.05 vs. si-Gm41664 + anti-miR-150. **D**–**F** MiR-150 knockdown reverses the anti-apoptotic effects of si-Gm41664 in CMs. CMs were transfected as indicated and subjected to in vitro simulation of I/R (hypoxia/reoxygenation) [sI/R (H/R)]. TUNEL assays were then conducted in both normoxic (**D**, **F**) and sI/R conditions (**E**, **F**). The percentage of apoptotic nuclei (green) was calculated after the normalization of total nuclei (blue). Scale bar = 100 μm. *N* = 6. One-way ANOVA with Tukey multiple comparison test. *P < 0.05, **P < 0.01, or ***P < 0.001 vs. control. ^#^*P* < 0.05, ^##^*P* < 0.01, or ^###^*P* < 0.001 vs. si-Gm41664 + anti-miR-150. All data are presented as mean ± SEM.

Since miR-150 negatively regulates CM apoptosis in vitro and in vivo [25, 26], we next examined whether the novel upstream regulator of miR-150, Gm41664 regulates CM apoptosis. We first find that Gm41664 knockdown in CMs decreases apoptotic *p53*, *Casp14*, and *Ing-4* while miR-150 knockdown increases the expression of these apoptotic genes (Fig. 7A–C and Fig. S8C, D). In agreement with this mRNA data, we also observe that CMs with Gm41664 knockdown exhibit enhanced protein levels of anti-apoptotic BCL2 and suppressed protein levels of apoptotic BAX and p53 (Fig. S9A–C). Caspase 3/7 luciferase activity in CMs is also decreased upon Gm41664 knockdown in basal and sI/R (H/R) conditions (Fig. S9D). Our loss-of-function studies also show that Gm41664 knockdown decreases CM apoptosis in basal and sI/R (H/R) conditions (Fig. 7D–F). This result indicates that Gm41664 is required for CM apoptosis. To directly interrogate a functional interaction between Gm41664 and miR-150 in CM apoptosis, we finally employed a siRNA/anti-miR-based rescue strategy to validate the functional relevance of Gm41664, the novel upstream regulator of miR-150. The anti-miR-150 treatment increases CM apoptosis (Fig. 7D–F), consistent with QRT-PCR data (Fig. 7C and Fig. S8C, D). Importantly, decreased CM apoptosis after Gm41664 knockdown is reversed by anti-miR against miR-150 (Fig. 7D–F). These TUNEL data agree with our QRT-PCR data, showing that miR-150 knockdown reverses the decreased levels of apoptotic genes (Fig. 7C and Fig. S8C, D) mediated by Gm41664 knockdown. Thus, these CM data along with our in vivo evidence indicate that Gm41664 is a critical negative regulator of CM miR-150.

### MiR-150 acts as a gatekeeper of human cardiomyocyte survival partially by decreasing apoptotic GDAP1L1

Due to miR-150’s downregulation in CMs exposed to sI/R (H/R) conditions [25], we next tested if the novel target of miR-150, *GDAP1L1* is inversely modulated in human CMs (HuCMs) subjected to sI/R (H/R) conditions. Indeed, we observe that *GDAP1L1* is increased in HuCMs after sI/R (H/R) (Fig. S10A), which agrees with our in vivo data in post-ISO mouse hearts (Fig. 5F). We also find that CM *GDAP1L1* is upregulated after miR-150 knockdown (Fig. S10B). These data indicate that *GDAP1L1* is an important functional target of CM miR-150 because miR-150 is decreased in sI/R (H/R) and MI [25], I/R [51, 52], as well as chronic catecholamine stimulation (Fig. 1A).

**Fig. 8:**
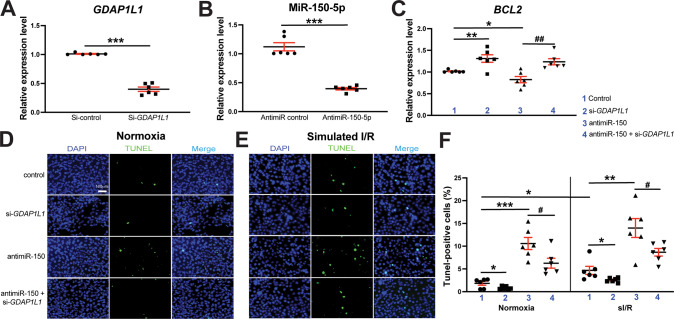
*GDAP1L1* is required for miR-150-dependent inhibition of human cardiomyocyte apoptosis. **A**, **B** AC16 cells were transfected with control scramble siRNA (si-control) or *GDAP1L1* siRNA (si-*GDAP1L1*) (**A**) and with anti-miR control scramble or anti-miR-150 (**B**). QRT-PCR for *GDAP1L1* (**A**) or miR-150 (**B**) was conducted to check the knockdown efficiency. Data were normalized to *GAPDH* (**A**) or *U6 SNRNA* (**B**) and expressed relative to controls. *N* = 6 per group. Unpaired two-tailed *t*-test. ***P < 0.001 vs. si-control or anti-miR control. **C** QRT-PCR expression analysis of anti-apoptotic *BCL2* in human cardiomyocytes (HuCMs) transfected with 4 different groups as indicated. *N* = 6. *BCL2* expression compared to *GAPDH* was calculated using 2^−ΔΔCt^, and data are presented as fold induction of *BCL2* expression levels normalized to control (si-control or anti-miR control). One-way ANOVA with Tukey multiple comparison test. *P < 0.05 or **P < 0.01 vs. control. ^##^*P* < 0.01 vs. anti-miR-150. **D**–**F** RNA interference with *GDAP1L1* protects HuCMs from the pro-apoptotic effects mediated by anti-miR-150. HuCMs were transfected as indicated and subjected to in vitro simulation of I/R (hypoxia/reoxygenation) [sI/R (H/R)]. TUNEL assays were then conducted in both normoxic (**D**, **F**) and sI/R conditions (**E**, **F**). Scale bar = 100 μm. The percentage of apoptotic nuclei (green) was calculated after the normalization of total nuclei (blue). *N* = 6. One-way ANOVA with Tukey multiple comparison test. *P < 0.05, **P < 0.01, or ***P < 0.001 vs. control. ^#^*P* < 0.05 vs. anti-miR-150. All data are shown as mean ± SEM.

We next examined whether the novel target of miR-150, *GDAP1L1* controls HuCM apoptosis. We first find that *GDAP1L1* knockdown in HuCMs decreases apoptotic *p53*, *BAK1*, and *BAX* in response to sI/R (H/R) conditions (Fig. 8A and Fig. S11), while increasing anti-apoptotic *BCL2* in normoxia (Fig. 8C). In agreement with this QRT-PCR data, we also observe that HuCMs transfected with siRNAs against *GDAP1L1* exhibit suppressed protein levels of apoptotic CLEAVED CASPASE-3, EGR2, p53, and BAX, while increasing anti-apoptotic BCL2 (Fig. S12). Our loss-of-function approaches also show that *GDAP1L1* knockdown decreases HuCM apoptosis in basal and sI/R (H/R) conditions (Fig. 8D–F). These results suggest that *GDAP1L1* is sufficient to promote HuCM apoptosis. To establish a functional link between miR-150 and *GDAP1L1* in HuCM apoptosis, we next applied an anti-miR/siRNA-based rescue strategy to validate the functional relevance of *GDAP1L1*. First, anti-miR-150 treatment increases *GDAP1L1* (Fig. S10B). MiR-150 knockdown also increases HuCM apoptosis, which is attenuated by siRNA against *GDAP1L1* (Fig. 8B, D–F). These TUNEL results agree with our QRT-PCR data, showing that miR-150 knockdown suppresses anti-apoptotic *BCL2*, which is attenuated by siRNA against *GDAP1L1* (Fig. 8C), and that miR-150 knockdown increases pro-apoptotic *p53* and *BAK1* (Fig. S10C, D). Lastly, we also show that Gm41664 knockdown decreases the expression in *Gdap1l1* in mouse CMs and reverses *Gdap1l1* upregulation mediated by miR-150 knockdown (Fig. S13). Altogether, these CM data along with our in vivo results indicate that CM miR-150 confers protective actions partially by the direct functional repression of apoptotic *Gdap1l1*, establishing the novel axis of Gm41664/miR-150/GDAP1L1 in CMs.

## Discussion

Here, we identify the functional link among Gm41664, miR-150, and *Gdap1l1* as a novel regulatory mechanism of β_1_AR/β-arrestin protective signaling pertinent to excess catecholamine stimulation. Using a novel DTG mouse model, we show that genetic overexpression of miR-150 relieves cardiac dysfunction mediated by CM-specific loss of β_1_AR/β-arrestin signaling. Mechanistically, we unveil that miR-150 directly suppresses apoptotic *Gdap1l1* and that Gm41664 blocks the inhibitory effect of miR-150 on *Gdap1l1* via its ceRNA action. Therefore, repressed expression of *Gdap1l1* in GRK^**–**^β_1_AR/miR-150 DTG mice causes improved cardiac function post-ISO, whereas increased expression of *GDAP1L1* in HuCMs lacking miR-150 causes elevated CM apoptosis.

The β_1_AR uses GRK5/6 and β-arrestins to promote CM survival against chronic catecholamine stimulation in the absence of G protein activation [12]. We reported that miR-150 is induced by the β-blocker Carv acting through the β_1_AR/β-arrestin protective signaling [14]. Along with the results presented here (Fig. S14), we suggest that miR-150 may be an important downstream mechanism by which β_1_AR/β-arrestin signaling pathways elicit protection, and that newly discovered β_1_AR/β-arrestin-mediated regulatory mechanisms of miR-150 activation in the current study confer beneficial remodeling in failing hearts by repressing CM apoptosis via direct suppression of apoptotic genes such as *Gdap1l1*.

We showed using a miR-150 KO mouse model that miR-150 is protective in MI by decreasing CM apoptosis [25]. We also demonstrated that CM-specific miR-150 cKO mice worsen apoptosis and adverse remodeling post-MI [26]. Another group reported that miR-150 overexpression protects the heart from acute MI by suppressing the monocyte migration [31]. Cardiac-specific overexpression of miR-150 was also shown to blunt TAC-induced HF [32]. Although there has been increasing data showing the importance of miR-150 in HF, direct evidence firmly establishing in vivo functional link between β_1_AR/β-arrestin signaling and miR-150 in HF is lacking, and mechanisms that regulate miR-150 expression and function in HF have not been adequately defined. In this study, we report novel discoveries that establish the in vivo functional relationship between β_1_AR/β-arrestin signaling selectively in CMs and miR-150 in HF, delineate the functional Gm41664/miR-150 link in CM apoptosis, as well as identify a new direct and functional target of miR-150, *Gdap1l1* in CMs.

We further discover that miR-150 controls a surprisingly small number of cardiac circRNAs, lncRNAs, and genes after ISO stimulation using genome-wide ncRNA and mRNA profiling analyses. We first find that CM-specific loss in β_1_AR/β-arrestin signaling inhibits cardiac circRNA-31100 and circRNA-32197 after chronic catecholamine stimulation, which is indirectly opposed by miR-150. We next observe that CM-specific deficiency in β_1_AR/β-arrestin signaling increases cardiac lncRNAs (Gm41664, Gmds, and Tspan2os) while decreasing cardiac lncRNA AK036033 after chronic catecholamine treatment. These lncRNA expression patterns are reversed by miR-150 overexpression. Lastly, dysregulated cardiac genes are enriched in both known and unknown pathways for miR-150, such as ARVC, autophagy, DCM, cell cycle, regulation of actin cytoskeleton, and MAPK signaling (Fig. S6D, Fig. S7C, and Table S5). Our further prediction, validation, and functional analyses lead us to discover that Gm41664 is a novel regulator of miR-150 and that *Gdap1l1* is a new direct and functional target of miR-150 in CMs.

Gm41664 is a natural anti-sense lncRNA of *Kif5b* and there are no reports on this lncRNA. Here, we report novel findings that Gm41664 expression is increased in CM-specific deficiency in β_1_AR/β-arrestin signaling after chronic ISO treatment, concurrent with its downregulation by miR-150 overexpression. Our CM functional analyses further show that Gm41664 increases CM apoptosis by regulating the novel miR-150/GDAP1L1 axis. GDAP1L1 is conserved in vertebrates and is involved in the mitochondrial fission [42]. However, nothing is known about the cardiovascular roles and regulatory mechanisms of GDAP1L1. Especially, whether GDAP1L1 can be regulated by the β_1_AR/β-arrestin/Gm41664/miR-150 axis, or if it can contribute to adverse cardiac cell function remains undetermined. Our novel data first show that cardiac *Gdap1l1* expression is increased in GRK^**–**^β_1_AR TG mice after chronic catecholamine stimulation (Fig. 5F), concurrent with Gm41664 upregulation (Fig. 4D) and miR-150 downregulation (Fig. 1A). Interestingly, our HuCM data also show that *GDAP1L1* is upregulated after sI/R (H/R) (Fig. S10A), indicating a critical role of *GDAP1L1* in CMs. In agreement with this idea, we demonstrate for the first time that *GDAP1L1* knockdown represses HuCM apoptosis (Fig. 8D–F and Fig. S11–12). Thus, our in vivo mouse hearts and in vitro HuCM findings support that inhibition of *Gdap1l1* may be an important therapeutic option for HF. Given our results that apoptotic *GDAP1L1* is a novel direct and functional target for miR-150 in CMs (Fig. 6C, D and Fig. 8), HF patients with repressed levels of miR-150 may be particularly considered for future targeted treatment strategies based on *GDAP1L1*.

In conclusion, our data using a novel gain-of-function mouse model suggest that genetic overexpression of miR-150 attenuates excessive cardiac dysfunction post-catecholamine mediated by CM-selective abrogation of β_1_AR/β-arrestin signaling. Our CM studies also indicate that miR-150 is protective partially by mitigating CM apoptosis via its direct functional suppression of apoptotic *Gdap1l1*, and that lncRNA Gm41664 opposes miR-150’s actions via a ceRNA mechanism. Although miR-150 is linked with HF in humans [33] and its correlative relationship with β_1_AR/β-arrestin protective signaling is shown [14], our studies using a novel mouse model and unbiased genome-wide profiling analyses directly establish in vivo functional relationship between β_1_AR/β-arrestin signaling and miR-150 in chronic catecholamine-induced HF, as well as discover the underlying mechanisms by which miR-150 suppresses myocardium apoptosis and is controlled in CMs. Given that miR-150 downregulation also underlies many forms of cardiac abnormalities [27, 51–53], the protective action of miR-150 may be commonly applied to various stress conditions. Therefore, boosting miR-150 levels by miR-150 overexpression or Carv, partially to blunt CM apoptosis, may be a valid adjunctive option for providing therapeutic benefits. Our current study also points to the urgent need for future studies that investigate the effects of gain- or loss-of-function of newly identified upstream (by Gm41664) and downstream (by GDAP1L1) mechanisms of miR-150 in HF.

## Materials and methods

### Animal study, treatment protocol, and ethics committee approval

We employed cardiac-specific TG mice overexpressing mutant β_1_ARs that lack GRK phosphorylation sites (GRK^−^β_1_AR TG) as described [12]. A CAG promoter-driven miR-150 TG mouse line [54] was kindly provided by Dr. Jennifer Richer at the University of Colorado, and was then bred to GRK^−^β_1_AR TG mice to generate the novel GRK^−^β_1_AR TG/miR-150 DTG mouse line. Mice were maintained on a C57BL/6 J background and 8–16-week-old mice of both sexes were used. Genotype- and sex-matched mice were randomly assigned to experimental groups mitigating the cage effect. Researchers were blinded to the genotype of the animals until the end of the analysis. The use of animals in the current study conformed to the Guidelines for the Care and Use of Laboratory Animals published by the US National Institutes of Health. All animal experiments were performed according to the protocols approved by the Institutional Animal Care and Use Committee at Indiana University (approval reference #21189). ISO (Sigma–Aldrich) was dissolved in 0.002% ascorbic acid with saline and mini-osmotic pumps (Alzet model 2001; DURECT Corporation) were then filled to deliver at the rate of 3 mg/kg/day for 7 days as previously optimized for GRK^−^β_1_AR TG [12]. A vehicle (0.002% ascorbic acid with saline) was administrated in control mice. Seven days after ISO administration, mice were euthanized by thoracotomy under 1–4% inhalant isoflurane anesthesia. LV tissues were then excised and flash-frozen in liquid N_2_ for downstream assays as we published [14].

### Cell culture and transfection

The immortalized mouse adult atria-derived CM HL-1 cell line obtained from Dr. Claycomb was maintained and used as previously described [55]. The AC16 adult human CM cell line was purchased from Sigma–Aldrich (SCC109) and maintained according to the company’s recommendation. The cell line was established from primary cells by transfecting the SV40 T antigen, and it displayed normal CM characteristics and cellular markers. We used multiple batches of the cell lines subjected to 3–5 passages in culture. Both HL-1 and AC16 CM cell lines were authenticated by the supplier, and they have been monitored regularly for their authenticity and to be negative for mycoplasma contamination. Using Lipofectamine™ 3000 reagent (Invitrogen) as previously described [13, 25], HL-1 cardiomyocytes were transfected with an Ambion Silencer^TM^ Select Negative Control siRNA (Thermo Fischer Scientific, 4390843) or an Ambion Silencer^TM^ Select Pre-Designed siRNA targeting mouse Gm41664 (Thermo Fischer Scientific, 4390771, siRNA ID: n431261). AC16 cardiomyocytes were also transfected with an ON-TARGETplus Non-targeting Negative Control siRNA (Horizon Discovery Ltd, D-001810-10-05) or an ON-TARGETplus GDAP1L1 siRNA-SMARTpool targeting human GDAP1L1 (Horizon Discovery Ltd, L-017342-01-0005, ID: 78997). To inhibit the expression of miR-150 in CMs, we transfected Ambion Anti-miR^TM^ miR inhibitors (Life Technologies) specific to miR-150 (MH10070) and a miR inhibitor negative control (4464076) using Lipofectamine™ 3000 reagent (Invitrogen) as described previously [56]. For gain-of-function studies, we transfected the cells with a miR mimic negative control (4464058) and miR-150 mirVana^TM^ mimic (Life Technologies, MC10070). All in vitro assays were performed 60–72 h after transfection when maximum knockdown efficiency was reached.

### In vitro simulated ischemia/reperfusion (hypoxia/reoxygenation) [sI/R (H/R)]

Cardiomyocytes plated on coverslips or six-well plates were transfected with miR inhibitors or siRNAs as aforementioned, washed, and incubated in a simulated ischemia buffer that contained 118 mM NaCl, 24 mM NaH_2_CO_3_, 1 mM NaHPO_4_, 2.5 mM CaCl_2_, 1.2 mM MgCl_2_, 20 mM sodium lactate, 16 mM KCl, and 10 mM 2-deoxyglucose (pH 6.2). Cardiomyocytes were then placed in the hypoxic chamber (5% CO_2_, 0.1% O_2_) for 3 h followed by replacing the ischemic buffer with a normal cell medium, and were incubated under normoxia conditions for 4 h to complete the sI/R protocol as described [18, 25]. Coverslips or plates were processed for QRT-PCR, immunoblotting, TUNEL staining, and caspase-Glo 3/7 assays.

### RNA isolation and real-time QRT-PCR analyses

Total RNA from CMs and mouse hearts was prepared using Trizol Reagent (Invitrogen) and treated with RNase-free DNase I to remove genomic DNA as described [57, 58]. For the detection of mature miR-150, the TaqMan MiR Reverse Transcription Kit (Life Technologies) was used to synthesize cDNA for TaqMan MiR Assays. We used the miR-150 TaqMan probe (000473; Life Technologies) to measure the evolutionarily conserved mature miR-150 by Real-Time RT-PCR. U6 snRNA probe, 001973 (Life Technologies) was used for an endogenous control.

cDNA for genes and ncRNAs was synthesized using SuperScript III reverse transcriptase (Invitrogen) and random hexamers. Expression of genes or lncRNAs was detected using TaqMan expression assays for the mouse (*Anp*, Mm00435329_m1; *Tnf-α*, Mm00443258_m1; *Col3a1*, Mm00802300_m1; *Bax*, Mm00432051_m1; *Bak1*, Mm00432045_m1; *Egr2*, Mm00456650_m1; *P2x7r*, Mm00440578_m1; *Casp14*, Mm00438040_g1; *Ing-4*, Mm00460097_m1; *p53*, Mm01731290_g1; *Gdap1l1*, Mm00523187_m1; *Cspg5*, Mm00516549_m1; *Cfl1*, Mm03057591_g1; *Mfsd12*, Mm01172867_m1; *Arhgef39*, Mm01349584_g1; *Map2k7*, Mm00488759_m1; *Cdk14*, Mm00448111_m1; *Slitrk6*, Mm07302106_m1; *Gm41664*, Mm03960618_m1; *Tspan2os*, Mm01305745_m1; *AK036033*, Mm01302617_m1, and *Gapdh*, Mm99999915_g1 for an endogenous control), and the human (*GDAP1L1*, Hs00225209_m1; *BCL2*, Hs04986394_s1; *BAK1*, Hs00832876_g1; *BAX*, Hs00180269_m1; *p53*, Hs01034249_m1, and *GAPDH*, Hs02786624_g1 for an endogenous control). Expression of circRNAs was detected using Circular RNA qPCR service (Arraystar Inc). Briefly, circRNAs primers were designed specifically for the circular junction sites to achieve highly specific and accurate detection of circRNAs, even in the presence of linear counterparts. The following sense and anti-sense primers were used to amplify and measure the amount of circRNAs by SYBR Green-based Real-Time RT-PCR: mmu_circRNA-31100, 5’-GCTCTGCCTATGAGTTCTACCA-3’ and 5’-TTTTCAGAGCAGAAGTTCTGGT-3’. mmu_circRNA-32197, 5’-TGCTGTGCTGCCTTAGACAGG-3’ and 5’-TCCGAGATGGAATGAAGCAACT-3’. *Gapdh* (5’-CACTGAGCAAGAGAGGCCCTAT-3’ and 5’-GCAGCGAACTTTATTGATGGTATT-3’) was used for an endogenous control.

Real-time PCR Reactions were amplified and analyzed in triplicate using a QuantStudio 3 Detection System (Life Technologies) as described previously [58]. PCR reaction conditions were as follows: Step 1: 50 °C for 2 min, Step 2: 95 °C for 10 min, Step 3: 40 cycles of 95 °C for 15 seconds followed by 60 °C for 1 min. Expression compared to endogenous controls was calculated using 2^−ΔΔCt^ and levels were normalized to control.

### Apoptosis by TUNEL staining

DNA fragmentation was detected in situ using TUNEL [59]. In brief, hearts or cells were incubated with proteinase K, and DNA fragments were labeled with fluorescein-conjugated dUTP using terminal deoxynucleotidyl transferase (Roche Diagnostics). The total number of nuclei was determined by manual counting of DAPI-stained blue nuclei in six random fields per slide or coverslip (original magnification, ×200). All TUNEL-positive green nuclei were then counted. Digital photographs of fluorescence were acquired with a Zeiss microscope (ApoTome.2; Carl Zeiss) and processed with Adobe Photoshop CC 2021.

### Caspase-Glo 3/7 assay

Caspase 3/7 activity in CMs was measured with the Caspase-Glo 3/7 Assay kit (G8090, Promega) for detecting apoptosis. Briefly, 10 μg of protein in a 50 μl total volume was mixed with 50 μl of caspase-Glo 3/7 reagent and incubated for 1 h at room temperature. Luminescence was measured with the Synergy LX FA Multi-Mode Microplate Reader (BioTek Instruments), with each CM sample run in triplicate as described [25, 60].

### Statistical analysis

Data are presented as mean ± SEM (unless noted otherwise in the figure legend) from independent experiments with different biological samples per group. Triplicate experiments were performed for all biochemical and cell biology studies. The number of in vitro biological samples per group was 6. The number of mouse samples per group was 3–18. The exact sample size for each experimental group/condition is given as a number in the figure/table legend. To ensure the robustness of the data and to allow the direct evaluation of the distribution of the data, we present graphical data as scatter/dot plots. Statistical significance was determined by unpaired two-tailed *t*-test for comparisons between two groups, one-way ANOVA with Tukey multiple comparison test for multiple groups, two-way ANOVA with Tukey multiple comparison test for comparisons between two groups with different treatments, and two-way repeated-measures ANOVA with Bonferroni post hoc test for two groups over time. The unpaired two-tailed *t*-test was based on assumed normal distributions. A *P*-value <0.05 was considered statistically significant. *P*-values are indicated as follows: * or ^#^*P* < 0.05, ** or ^##^*P* < 0.01, and *** or ^###^*P* < 0.001.

## Supplementary information

Combined Supplementary Information

Table S3_CircRNAs Dataset

Table S4_LncRNAs Dataset

Table S5_Genes Dataset

Original Western Blot Images

## Data Availability

The microarray data discussed in this study have been deposited in NCBI’s Gene Expression Omnibus. Datasets are accessible through GEO Series access number GSE199290 for circRNA profiling, and GSE199195 for lncRNAs and mRNAs. All other data that are not included in this publication, analytical methods, and study materials will be made available to other researchers for purposes of reproducing results or replicating procedures. Other methods and full and uncropped western blot images are also provided in Supplementary Information.
