# Supplementary material for: MiR-150 blunts cardiac dysfunction in mice with cardiomyocyte loss of β1-adrenergic receptor/β-arrestin signaling and controls a unique transcriptome
Source: Cell Death Discov. 2022 Dec 30;8:504. doi: 10.1038/s41420-022-01295-9 (PMC9803679; doi:10.1038/s41420-022-01295-9)
Supplement: Supplementary file 1 — Combined Supplementary Information [file 41420_2022_1295_MOESM1_ESM.pdf]

Supplementary Information

Supplementary Results

Supplementary Materials & Methods

Supplementary References

2 Supplementary Tables

14 Supplementary Figures and Legends

Supplementary Spreadsheets

Table S3_CircRNAs.xls

Table S4_LncRNAs.xls

Table S5_Genes.xlsx

Full and uncropped original blot images

Supplementary Results

MiR-150 overexpression in mice with CM-specific deficiency in β-arrestin-mediated β_1AR signaling modulates the expression of a relatively small number of cardiac circular RNAs after chronic catecholamine stimulation

Among 14,236 mouse circRNAs that we profiled, 14 circRNAs are significantly upregulated, and 3 circRNAs are significantly downregulated (Figure S1A, Figure S2A and Table S3; see Up & Down_Vehicle DTG vs GRK sheet) in vehicle GRK$^-\beta_1$AR/miR-150 DTG compared to vehicle GRK$^-\beta_1$AR TG. We also find that 42 circRNAs are significantly upregulated, and 18 circRNAs are significantly downregulated (Figure S1B, Figure S2B and Table S3; see Up & Down_GRK$^-\beta_1$AR ISO vs Vehicle sheet) in ISO GRK$^-\beta_1$AR TG compared to vehicle GRK$^-\beta_1$AR TG. In addition, 58 circRNAs are significantly upregulated, and 20 circRNAs are significantly downregulated (Figure S1C, Figure S2C and Table S3; see Up & Down_DTG ISO vs Vehicle sheet) in ISO GRK$^-\beta_1$AR/miR-150 DTG compared to vehicle GRK$^-\beta_1$AR/miR-150 DTG. Lastly, we observe that 458 circRNAs are significantly upregulated, and 517 circRNAs are significantly downregulated (Figure S1D, Figure S2D and Table S3; see Up & Down_ISO DTG vs GRK sheet) in ISO GRK$^-\beta_1$AR/miR-150 DTG compared to ISO GRK$^-\beta_1$AR TG.

MiR-150 overexpression in mice with CM-specific loss of β-arrestin-mediated β_1AR signaling induces unique cardiac long noncoding RNA signatures post-chronic catecholamine stimulation

Among 35,923 profiled mouse lncRNAs, 332 lncRNAs are significantly upregulated, and 79 lncRNAs are significantly downregulated (Figure S4A and Table S4; see Up & Down_Vehicle

DTG vs GRK sheet) in vehicle GRK$^-\beta_1$AR/miR-150 DTG compared to vehicle GRK$^-\beta_1$AR TG. We also observe that 118 lncRNAs are significantly upregulated, and 67 lncRNAs are significantly downregulated (Figure S4B and Table S4; see Up & Down_GRK$^-\beta_1$AR ISO vs Vehicle sheet) in ISO GRK$^-\beta_1$AR TG compared to vehicle GRK$^-\beta_1$AR TG. In addition, 286 lncRNAs are significantly upregulated, whereas 364 lncRNAs are significantly downregulated (Figure S4C and Table S4; see Up & Down_DTG ISO vs Vehicle sheet) in ISO GRK$^-\beta_1$AR/miR-150 DTG compared to vehicle GRK$^-\beta_1$AR/miR-150 DTG. Lastly, we find that 3,036 lncRNAs are significantly upregulated, whereas 1,848 lncRNAs are significantly downregulated (Figure S4D and Table S4; see Up & Down_ISO DTG vs GRK sheet) in ISO GRK$^-\beta_1$AR/miR-150 DTG compared to ISO GRK$^-\beta_1$AR TG.

MiR-150 overexpression in mice with CM-specific abrogation of β-arrestin-mediated β_1AR signaling regulates the expression of a subset of cardiac genes after excessive catecholamine stimulation

Among 24,881 profiled mouse genes, 143 genes are significantly upregulated, and 75 genes are significantly downregulated (Figure S5A and Table S5; see Up & Down_Vehicle DTG vs GRK sheet) in vehicle DTG compared to vehicle GRK$^-\beta_1$AR TG. We also observe that 66 genes are significantly upregulated and 37 genes are significantly downregulated (Figure S5B and Table S5; see Up & Down_GRK$^-\beta_1$AR ISO vs Vehicle sheet) in ISO GRK$^-\beta_1$AR TG compared to vehicle GRK$^-\beta_1$AR TG. In addition, 177 genes are significantly upregulated, and 233 genes are significantly downregulated (Figure S5C and Table S5; see Up & Down_DTG ISO vs Vehicle sheet in ISO DTG compared to vehicle DTG) in ISO GRK$^-\beta_1$AR/miR-150 DTG compared to vehicle GRK$^-\beta_1$AR/miR-150 DTG. Lastly, we find that 1,878 genes are significantly upregulated, and 1,453 genes are significantly downregulated (Figure S5D and Table S5; see Up & Down_ISO DTG vs GRK sheet) in ISO DTG compared to ISO GRK$^-\beta_1$AR TG.

To delineate the functional roles of differentially regulated genes in GRK$^{-}\beta_1$AR TG and GRK$^{-}\beta_1$AR/miR-150 DTG mice, we then categorized differentially expressed genes via the signaling pathway classification system of Kyoto Encyclopedia of Genes and Genomes (KEGG). Our signaling pathway analyses in vehicle DTG compared to vehicle GRK$^{-}\beta_1$AR TG demonstrate that upregulated genes are involved in EGFR tyrosine kinase inhibitor resistance, longevity regulation pathway, and HIF-1 signaling pathway (Figure S6A). In contrast, downregulated genes are enriched to Rap1 signaling pathway, regulation of actin cytoskeleton, and Ras signaling pathway (Figure S7A). We also observe that the top canonical signaling pathways for upregulated genes in ISO GRK$^{-}\beta_1$AR TG compared to vehicle GRK$^{-}\beta_1$AR TG include aldosterone synthesis and secretion, morphine addiction, and chemokine signaling pathway (Figure S6B). In addition, the expression of genes involved in complement and coagulation cascades, and Hippo signaling pathway are significantly upregulated (Figure S6C), whereas the expression of genes involved in ubiquitin- mediated proteolysis are significantly downregulated (Figure S7B) in ISO DTG compared to vehicle DTG. Lastly, we find that genes involved in arrhythmogenic right ventricular cardiomyopathy (ARVC), autophagy and dilated cardiomyopathy (DCM) are significantly increased (Figure S6D) in ISO DTG compared to ISO GRK$^{-}\beta_1$AR TG. In contrast, genes involved in cell cycle, regulation of actin cytoskeleton, and MAPK signaling pathway are significantly decreased (Figure S7C). Taken together, our gene profiling results suggest that miR-150 in mice with CM-specific loss of β-arrestin-mediated β_1AR signaling alters a subset of cardiac genes/signaling pathways to decrease cardiac dysfunction.

Supplementary Materials & Methods

Transthoracic echocardiography

Left ventricular (LV) performance was assessed by two-dimensional echocardiography using a Vevo 2100 Ultrasound (Visual Sonics) at baseline (pre-surgery) and post-isoproterenol (ISO) at 7 days as previously published [1, 2]. M-mode tracings were used to measure anterior and posterior wall thicknesses at end-diastole and end-systole. Parameters including LV internal diameter (LVID) in either diastole (LVIDd) or systole (LVIDs), end-diastolic volume (EDV), and end-systolic volume (ESV) were obtained. A single operator blinded to mouse genotypes performed echocardiography and data analysis. Fractional shortening (FS) was calculated according to the following formula: FS (%) = [(LVIDd-LVIDs)/LVIDd] X 100. Ejection fraction (EF) was calculated by: EF (%) = [(EDV-ESV)/EDV] X 100. All other LV performance parameters were also obtained as shown in Table S1-2.

Histopathological and immunohistochemical analyses

Morphometric analyses of heart size and weights were performed as we reported previously [1-3]. Histopathological analyses of myocardial tissues such as fibrosis by Masson's trichrome staining were conducted as previously described [4, 5]. For gross histological assessment, heart sections were stained with hematoxylin and eosin (H&E). Cardiac sections were also stained for TUNEL to assess apoptosis using *In Situ* Cell Death Detection Kit (Sigma-Aldrich) as we published [6].

Genome-wide circular RNA, long noncoding RNA and mRNA profiling analyses

Total RNAs from 12 independent mouse left ventricles (3 vehicle GRK$^{-}\beta_1$AR TG, 3 vehicle

$GRK^{-}\beta_1AR$;miR-150 DTG, 3 ISO $GRK^{-}\beta_1AR$ TG and 3 ISO $GRK^{-}\beta_1AR$;miR-150 DTG samples) were prepared as published [7]. RNA quantity and quality were assessed by the Synergy LX FA Multi-Mode Microplate Reader (BioTek Instruments). For circular RNAs (circRNAs), total RNAs were digested with Rnase R (Epicentre, Inc.) to remove linear RNAs and enrich circular RNAs. The DNA microarray experiments were then performed using the Mouse Circular RNA Microarray v2.0 (8 x 15K, Arraystar Inc) designed for the global profiling of 14,236 circular RNAs (circRNAs), as well as the Mouse LncRNA Microarray v3.0 (8 x 60K, Arraystar Inc) designed for the global profiling of 35,923 long noncoding RNAs (lncRNAs) and 24,881 protein-coding transcripts as previously described [7-11]. Fifteen positive probes for housekeeping genes and 20 negative probes were also included in the array as hybridization quality controls.

Labeling and array hybridization

RNA labeling and array hybridization were conducted according to the One-Color Microarray-Based Gene Expression Analysis protocol (Agilent Technology) as previously described [7, 12]. The Quick Amp Labeling Kit (Agilent Technologies) was used for sample labeling, and hybridization was performed in the SureHyb Hybridization Chamber (Agilent Technologies). Briefly, mRNAs were purified by using mRNA-ONLY™ Eukaryotic mRNA Isolation Kit (Epicentre Biotechnologies). Each sample was then amplified and transcribed into fluorescent cRNAs by utilizing a random priming method (Arraystar Super RNA Labeling Kit; Arraystar Inc). Next, the labeled cRNAs were purified by RNeasy Mini Kit (Qiagen). The concentration and specific activity of the labeled cRNAs (pmol Cy3/μg cRNA) were measured by NanoDrop ND-1000. 1 μg of each labeled cRNA was then fragmented by adding 5 μl 10 × Blocking Agent and 1 μl of 25 × Fragmentation Buffer. Then, the mixture was heated at 60°C for 30 minutes and 25 μl of 2 × Hybridization buffer was added to dilute the labeled cRNA. Lastly, 50 μl of hybridization solution was dispensed into the gasket slide and assembled into microarray slides. The slides were then incubated for 17 hours at 65°C in a hybridization oven (Agilent Technologies). The

hybridized arrays were washed, fixed and scanned by using the DNA Microarray Scanner G2505C (Agilent Technologies).

Analysis of microarray dataset

The Feature Extraction software version 11.0.1.1 (Agilent Technologies) was used to analyze acquired array images as described [7]. We performed more robust quantile normalization across all 12 samples than normalizing with one/some control probes (*i.e.,* the entire intensity distributions were normalized across the arrays, not just some reference points). The subsequent data processing was then performed using the R software limma package and the GeneSpring GX version 12.1 software package (Agilent Technologies). We then chose transcripts, in which at least 6 out of 12 samples exhibited flags in Present or Marginal, for further data analyses. We identified differentially expressed (DE) transcripts with statistical significance through Volcano Plot filtering between groups. For annotation for circRNA/miR interaction, the circRNA/miR interaction was predicted with Arraystar's home-made miR target prediction software. For protein-coding genes, we performed the pathway analysis for DE genes to identify the biological pathway by utilizing the GeneSpring Software GX version 12.1 (Agilent Technologies) and the latest Kyoto Encyclopedia of Genes and Genomes (KEGG; http://www.genome.jp/kegg) database. We used Fisher's exact test to calculate the *P* values of the Pathway ID and converted them to enrichment score by negative log10 transformation.

Immunoblotting, antibody, and detection

Cardiomyocytes were washed once with 1X PBS, solubilized in 1ml of lysis buffer (5mM HEPES, 250mM NaCl, 10% glycerol, 0.5% Nonidet P-40, 2mM EDTA, and protease inhibitors) for whole cell lysates as previously described [13]. Lysate samples were resolved by SDS-PAGE and transferred to PVDF (Bio-Rad) for immunoblotting. BCL-2 (3498, Rabbit, Cell Signaling), BAX (2772, Rabbit, Cell Signaling), p53 (sc-126, mouse, Santa Cruz), CLEAVED CASPASE-3 (9661,

Rabbit, Cell Signaling], EGR2 (AV100880, Rabbit, Sigma-Aldrich), GDAP1L1 (TA503153, Mouse, Thermo Fischer) and β-ACTIN (A5441, mouse, Sigma-Aldrich) primary antibodies were purchased and used at dilutions of 1:1,000 each. Detection was carried out using ECL (Amersham Biosciences).

In silico miR target prediction analyses and luciferase reporter-based miR-150 targeting assays

We used several prediction algorithms including miRwalk, miRanda, DIANA-microT-CDS, and Targetscan [14-16]. Each of these algorithms predicts hundreds of possible targets for miR-150. The mouse *Gm41664* and human *GDAP1L1* regions with the miR-150 binding site were cloned into the pmirGLO Dual-Luciferase miR Target Expression Vector (E1130, Promega). The following oligonucleotide pairs were designed, annealed, and ligated into the pmirGLO Vector to generate WT and mutant luciferase constructs. *Gm41664*: WT forward primer, 5'-AAACTAGCGGCCGCTAGTACATATCTGAAGTGTT***GGGAG***TT-3', WT reverse primer, 5'-CTAGAA***CTCCC***AACACTTCAGATATGTACTAGCGGCCGCTAGTTT-3', mutant forward primer, 5'-AAACTAGCGGCCGCTAGTACATATCTGAAGTGTT***TAAG***TT-3', and mutant reverse primer, 5'-CTAGAA***CTTA***AACACTTCAGATATGTACTAGCGGCCGCTAGTTT-3'. *GAP1L1*: WT forward primer, 5' AAACTAGCGGCCGCTAGTATAATCCCAGTGCTTT***GGGAG***AT-3', WT reverse primer, 5'-CTAGAT***CTCCC***AAAGCACTGGGATTATACTAGCGGCCGCTAGTTT-3', mutant forward primer, 5'-AAACTAGCGGCCGCTAGTATAATCCCAGTGCTTT***TAAG***AT-3', and mutant reverse primer, 5'-CTAGAT***CTTA***AAAGCACTGGGATTATACTAGCGGCCGCTAGTTT-3'.

Inserts and insertion sites were confirmed by ~140bp inserts when digested with NotI due to NotI sites in the vector and oligonucleotides, followed by sequencing for all plasmids. AC16 cells were transfected in 96-well plates with 0.1 μg of one of the luciferase plasmids along with either 50nM of miR mimic control or miR-150 mimic (MC10070; Life Technologies). At 72 hours after transfection, freshly collected cells were used for luciferase activities in Firefly and Renilla

buffers measured by Dual-Glo Luciferase Assay System (E2920, Promega) with the Synergy LX FA Multi-Mode Microplate Reader (BioTek Instruments) as described [1, 3].

Supplementary References

1. Bayoumi AS, Teoh JP, Aonuma T, Yuan Z, Ruan X, Tang Y, et al. MicroRNA-532 protects the heart in acute myocardial infarction, and represses prss23, a positive regulator of endothelial-to-mesenchymal transition. Cardiovasc Res. 2017; 113: 1603-14.

2. Bayoumi AS, Park KM, Wang Y, Teoh JP, Aonuma T, Tang Y, et al. A carvedilol-responsive microRNA, miR-125b-5p protects the heart from acute myocardial infarction by repressing pro-apoptotic bak1 and klf13 in cardiomyocytes. J Mol Cell Cardiol. 2017; 114: 72-82.

3. Tang Y, Wang Y, Park KM, Hu Q, Teoh JP, Broskova Z, et al. MicroRNA-150 protects the mouse heart from ischaemic injury by regulating cell death. Cardiovasc Res. 2015; 106: 387-97.

4. Ramakrishna S, Kim IM, Petrovic V, Malin D, Wang IC, Kalin TV, et al. Myocardium defects and ventricular hypoplasia in mice homozygous null for the Forkhead Box M1 transcription factor. Dev Dyn. 2007; 236: 1000-13.

5. Kim IM, Ackerson T, Ramakrishna S, Tretiakova M, Wang IC, Kalin TV, et al. The Forkhead Box m1 transcription factor stimulates the proliferation of tumor cells during development of lung cancer. Cancer Res. 2006; 66: 2153-61.

6. Park KM, Teoh JP, Wang Y, Broskova Z, Bayoumi AS, Tang Y, et al. Carvedilol-responsive microRNAs, miR-199a-3p and -214 protect cardiomyocytes from simulated ischemia-reperfusion injury. Am J Physiol Heart Circ Physiol. 2016; 311: H371-83.

7. Teoh JP, Park KM, Broskova Z, Jimenez FR, Bayoumi AS, Archer K, et al. Identification of gene signatures regulated by carvedilol in mouse heart. Physiol Genomics. 2015; 47: 376-85.

8. Zhu H, Han C, Lu D, Wu T. MiR-17-92 cluster promotes cholangiocarcinoma growth: evidence for PTEN as downstream target and IL-6/Stat3 as upstream activator. Am J Pathol. 2014; 184: 2828-39.

9. Cossette SM, Gastonguay AJ, Bao X, Lerch-Gaggl A, Zhong L, Harmann LM, et al. Sucrose non-fermenting related kinase enzyme is essential for cardiac metabolism. Biol Open. 2014; 4: 48-61.

10. Liao JM, Zeng SX, Zhou X, Lu H. Global effect of inauhzin on human p53-responsive transcriptome. PLoS ONE. 2012; 7: e52172.

11. Stueckle TA, Lu Y, Davis ME, Wang L, Jiang BH, Holaskova I, et al. Chronic occupational exposure to arsenic induces carcinogenic gene signaling networks and neoplastic transformation in human lung epithelial cells. Toxicol Appl Pharmacol. 2012; 261: 204-16.

12. Patterson TA, Lobenhofer EK, Fulmer-Smentek SB, Collins PJ, Chu TM, Bao W, et al. Performance comparison of one-color and two-color platforms within the MicroArray Quality Control (MAQC) project. Nat Biotechnol. 2006; 24: 1140-50.

13. Kim IM, Tilley DG, Chen J, Salazar NC, Whalen EJ, Violin JD, et al. Beta-blockers alprenolol and carvedilol stimulate beta-arrestin-mediated EGFR transactivation. Proc Natl Acad Sci U S A. 2008; 105: 14555-60.

14. Wang X. miRDB: a microRNA target prediction and functional annotation database with a wiki interface. RNA. 2008; 14: 1012-7.

15. Krek A, Grun D, Poy MN, Wolf R, Rosenberg L, Epstein EJ, et al. Combinatorial microRNA target predictions. Nat Genet. 2005; 37: 495-500.

16. Lewis BP, Shih IH, Jones-Rhoades MW, Bartel DP, Burge CB. Prediction of mammalian microRNA targets. Cell. 2003; 115: 787-98.

Figure S1

CircRNA Profiling

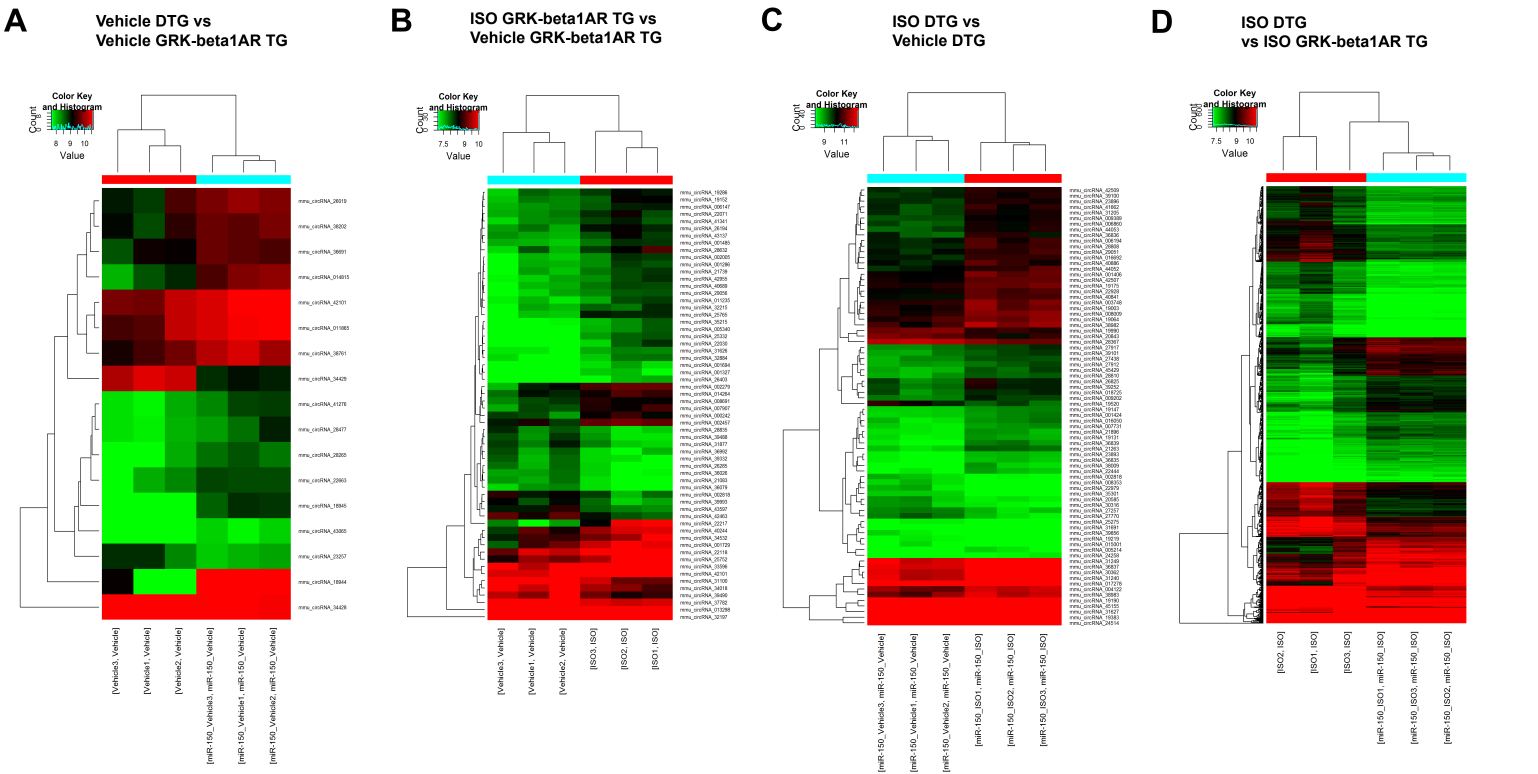

Figure S1. Circular RNA array analysis in mouse hearts. GRK$^{-}\beta_1$AR TG and GRK$^{-}\beta_1$AR;miR-150 DTG mice were infused with 0.002% ascorbic acid with saline (vehicle control) and isoproterenol (ISO) [3mg/kg/day] for 7 days by using micro-osmotic pumps. Microarray experiments (Arraystar Inc) were conducted in mouse hearts. The heat map represents circular RNA (circRNA) expression values in four groups. The expression level was visualized via colors. Green means that those circRNAs are downregulated in two compared groups. Red means that those circRNAs are upregulated in two compared groups. Black means no change in expression. The identified circRNAs are statistically significant among the 14,236 mouse circRNAs profiled.

Figure S2

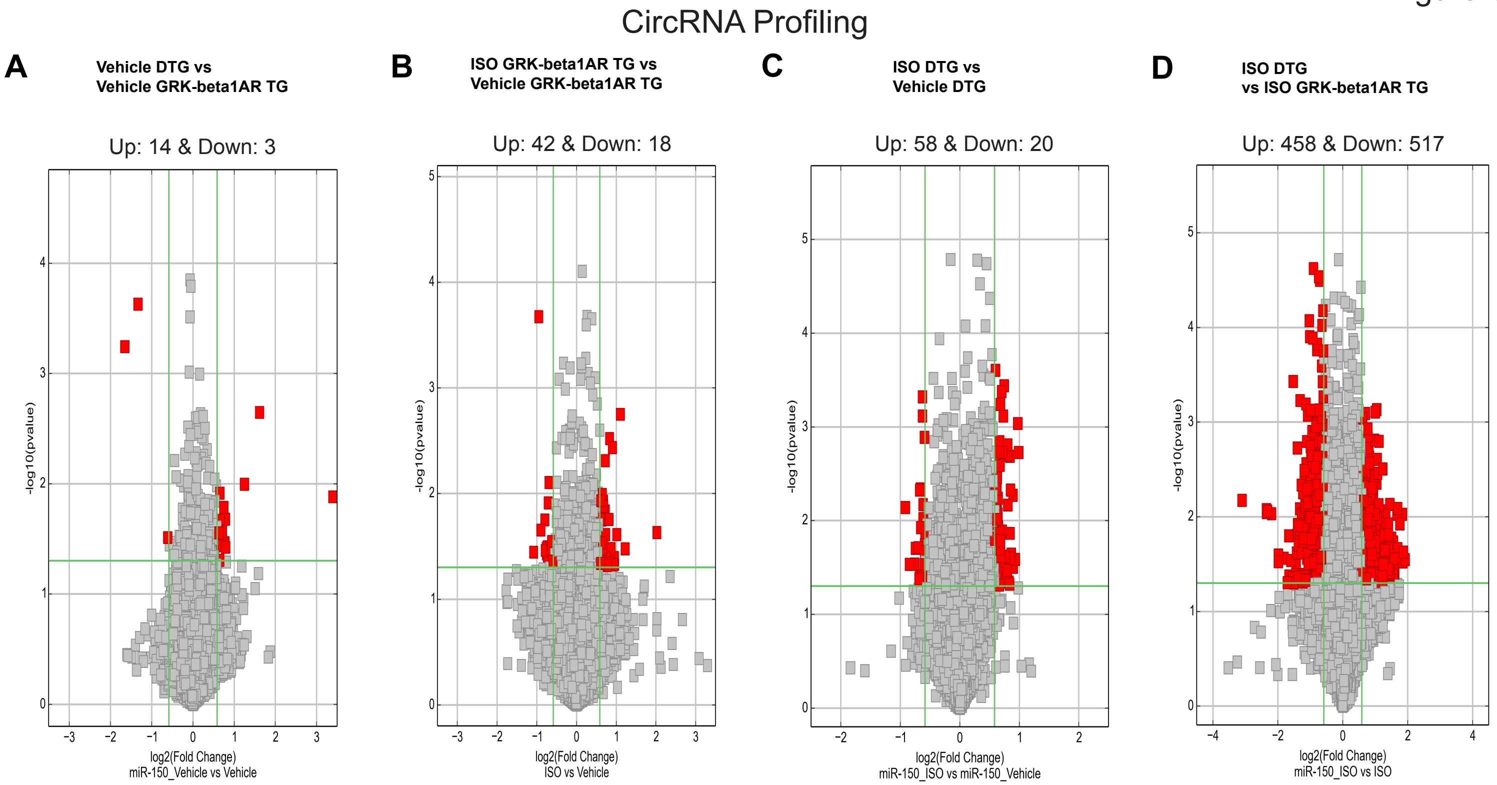

CircRNA Profiling
A
Vehicle DTG vs
Vehicle GRK-beta1AR TG
Up: 14 & Down: 3
-log10(pvalue)
log2(Fold Change)
miR-150_Vehicle vs Vehicle
B
ISO GRK-beta1AR TG vs
Vehicle GRK-beta1AR TG
Up: 42 & Down: 18
-log10(pvalue)
log2(Fold Change)
ISO vs Vehicle
C
ISO DTG vs
Vehicle DTG
Up: 58 & Down: 20
-log10(pvalue)
log2(Fold Change)
miR-150_ISO vs miR-150_Vehicle
D
ISO DTG
vs ISO GRK-beta1AR TG
Up: 458 & Down: 517
-log10(pvalue)
log2(Fold Change)
miR-150_ISO vs ISO

Figure S2. Volcano plot analyses of differentially expressed cardiac circular RNAs to discover circular RNA signatures that are regulated by β_1AR/β-arrestin-mediated signaling selectively in cardiomyocytes and modulate miR-150. A-D, Volcano Plots are made using fold-change values and *P* values that allow visualization of the relationship between fold-change and statistical significance, taking both magnitudes of change and variability into consideration. The vertical lines correspond to 1.5-fold up and down, and the horizontal line represents a *P* value of 0.05. So, the right (up) or left (down) points in the plot represent the differentially expressed (DE) circular RNAs (circRNAs) with statistical significance. DE circRNAs in vehicle $GRK^-\beta_1$AR;miR-150 DTG compared to vehicle $GRK^-\beta_1$AR TG controls are shown in **A**, DE circRNAs in ISO $GRK^-\beta_1$AR TG compared to vehicle $GRK^-\beta_1$AR TG controls are shown in **B**, DE circRNAs in ISO $GRK^-\beta_1$AR;miR-150 DTG compared to vehicle $GRK^-\beta_1$AR;miR-150 DTG are shown in **C**, and DE circRNAs in ISO $GRK^-\beta_1$AR;miR-150 DTG compared to ISO $GRK^-\beta_1$AR TG at 7 days post-treatment are shown in **D.**

Figure S3

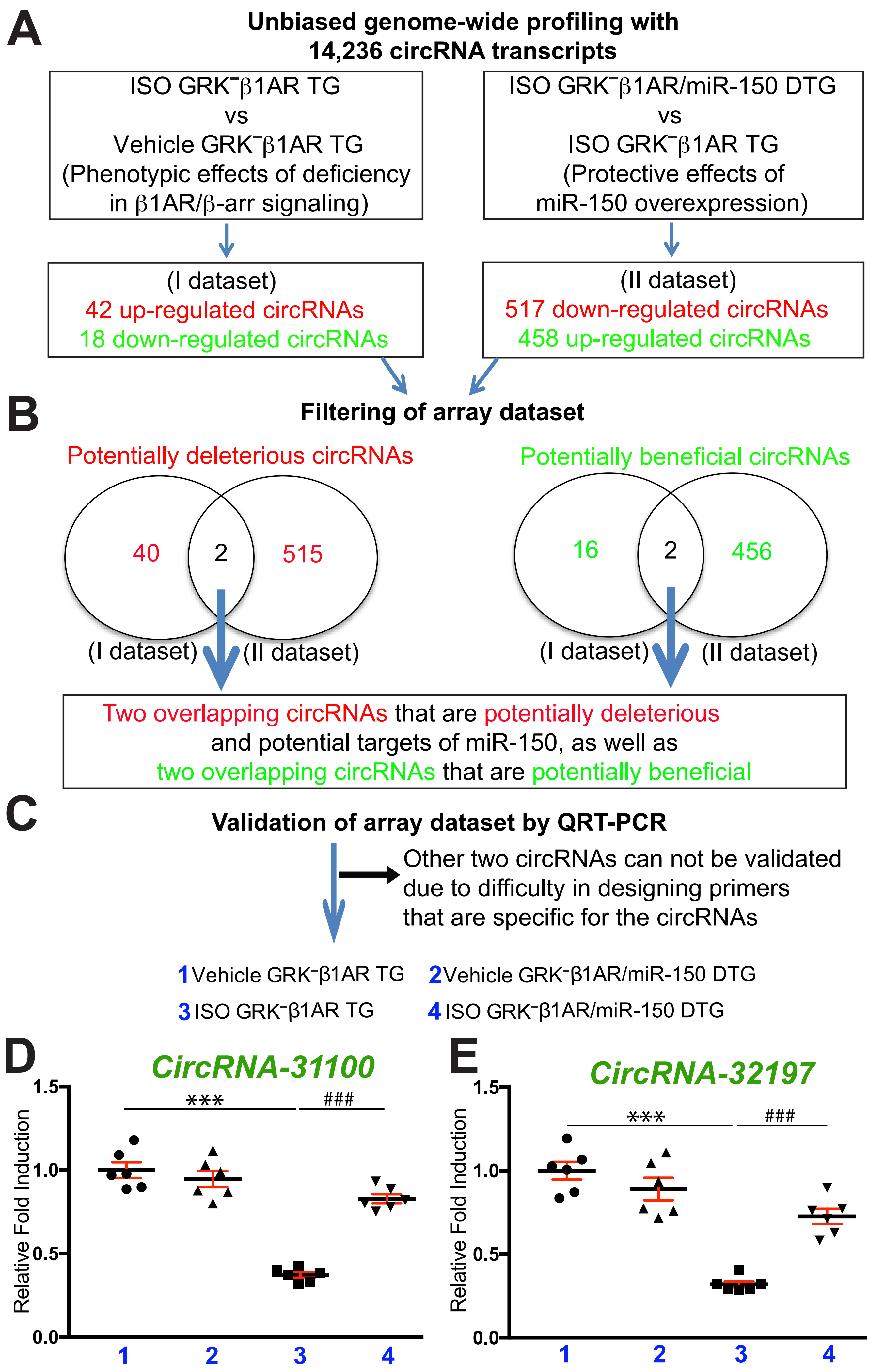
A
Unbiased genome-wide profiling with
14,236 circRNA transcripts
ISO GRK⁻β1AR TG
vs
Vehicle GRK⁻β1AR TG
(Phenotypic effects of deficiency
in β1AR/β-arr signaling)
ISO GRK⁻β1AR/miR-150 DTG
vs
ISO GRK⁻β1AR TG
(Protective effects of
miR-150 overexpression)
(I dataset)
42 up-regulated circRNAs
18 down-regulated circRNAs
(II dataset)
517 down-regulated circRNAs
458 up-regulated circRNAs
B
Filtering of array dataset
Potentially deleterious circRNAs
40
2
515
(I dataset)
(II dataset)
Potentially beneficial circRNAs
16
2
456
(I dataset)
(II dataset)
Two overlapping circRNAs that are potentially deleterious
and potential targets of miR-150, as well as
two overlapping circRNAs that are potentially beneficial
C
Validation of array dataset by QRT-PCR
Other two circRNAs can not be validated
due to difficulty in designing primers
that are specific for the circRNAs
1 Vehicle GRK⁻β1AR TG
2 Vehicle GRK⁻β1AR/miR-150 DTG
3 ISO GRK⁻β1AR TG
4 ISO GRK⁻β1AR/miR-150 DTG
D
CircRNA-31100
Relative Fold Induction
1.5
1.0
0.5
0.0
***
###
1
2
3
4
E
CircRNA-32197
Relative Fold Induction
1.5
1.0
0.5
0.0
***
###
1
2
3
4

Figure S3. Genome-wide circular RNA profiling in GRK$^{-}\beta_1$AR TG and GRK$^{-}\beta_1$AR;miR-150 DTG mice identifies novel circular RNAs that are regulated by β_1AR/β-arrestin-mediated signaling and modulate miR-150. A-B, Genome-wide profiling and filtering strategies of array dataset based on the correlation between transcript signatures and cardiac phenotypes. Two dysregulated (DE) circular RNAs (circRNAs), which are up-regulated in I dataset (ISO GRK$^{-}\beta_1$AR TG compared to vehicle GRK$^{-}\beta_1$AR TG controls) but are down-regulated in II dataset (ISO GRK$^{-}\beta_1$AR;miR-150 DTG compared to ISO GRK$^{-}\beta_1$AR TG) at 1 week post-treatment, were chosen for further analyses. Two other DE circRNAs, which are down-regulated in I dataset (ISO GRK$^{-}\beta_1$AR TG compared to vehicle GRK$^{-}\beta_1$AR TG controls) but are up-regulated in II dataset (ISO GRK$^{-}\beta_1$AR;miR-150 DTG compared to ISO GRK$^{-}\beta_1$AR TG) at 1 week post-treatment, were chosen for further analyses. N=3 per group. **C-D,** Validation strategy of array dataset. Two potentially beneficial DE circRNAs (CircRNA-31100 and CircRNA-32197) were validated by QRT-PCR analyses in LVs from GRK$^{-}\beta_1$AR TG and GRK$^{-}\beta_1$AR;miR-150 DTG mice at 1 week post-treatment. Note that other two circRNAs cannot be validated because of difficulty in designing primers specific for the circRNAs. We tried at least 4 different primer sets for each circRNA, but we failed to get reliable data due to wrong PCR product length on gel image, two peaks in melting curve, or no CT value. Data are presented as fold induction of circRNA expression normalized to *Gapdh*. N=6 per group. Two-way ANOVA with Tukey multiple comparison test. ***P<0.001 vs. vehicle; $^{\#\#\#}P$<0.001 vs. ISO GRK$^{-}\beta_1$AR TG. All data are presented as mean ± SEM.

Figure S4

LncRNA Profiling

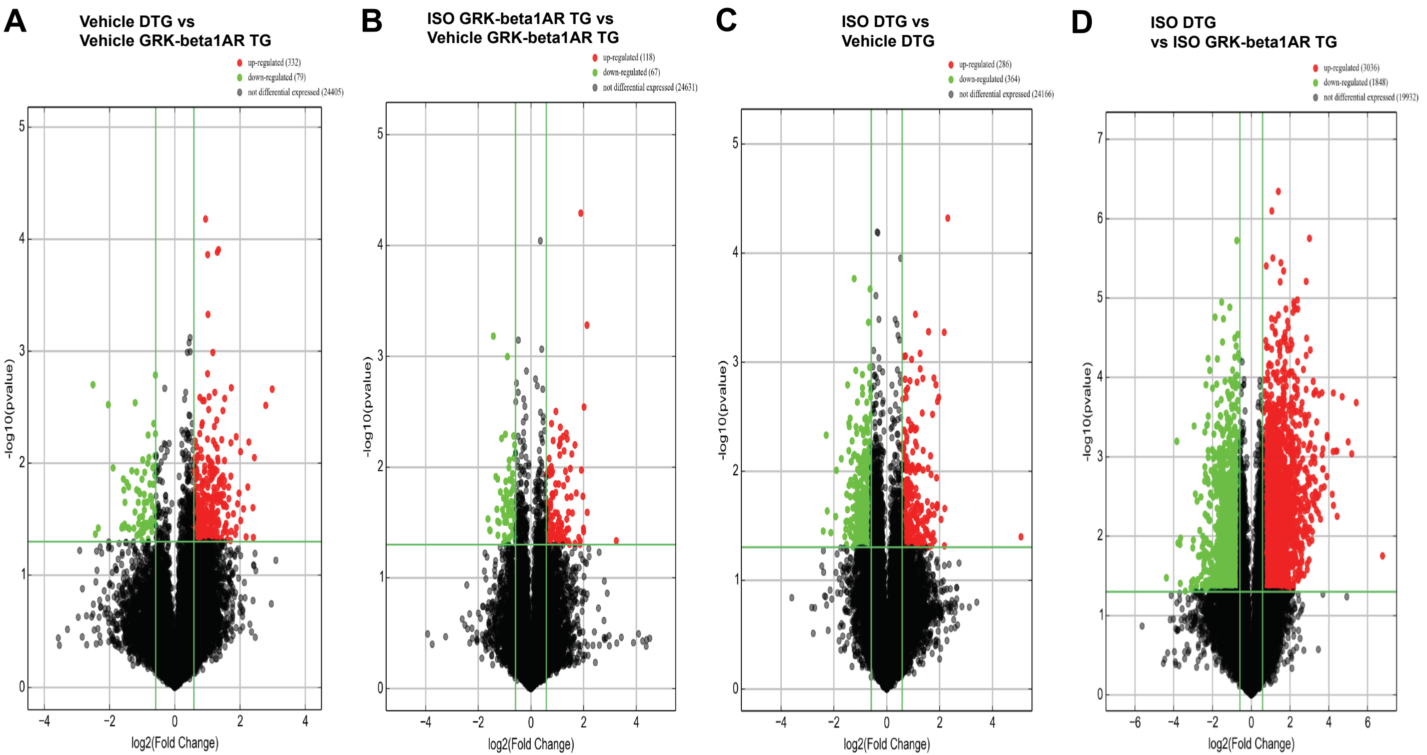

Figure S4. Volcano plot analyses of differentially expressed cardiac long noncoding RNAs to discover long noncoding RNA signatures that are controlled by β_1AR/β-arrestin-mediated signaling selectively in cardiomyocytes and regulate miR-150. A-D, Volcano Plots are made using fold-change values and *P* values that allow visualization of the relationship between fold-change and statistical significance, taking both magnitudes of change and variability into consideration. The vertical lines correspond to 1.5-fold up and down, and the horizontal line represents a *P* value of 0.05. So, the red (up) or green (down) points in the plot represent the differentially expressed (DE) long noncoding RNAs (lncRNAs) with statistical significance. DE lncRNAs in vehicle GRK$^{-}\beta_1$AR;miR-150 DTG compared to vehicle GRK$^{-}\beta_1$AR TG controls are shown in **A**, DE lncRNAs in ISO GRK$^{-}\beta_1$AR TG compared to vehicle GRK$^{-}\beta_1$AR TG controls are shown in **B**, DE lncRNAs in ISO GRK$^{-}\beta_1$AR;miR-150 DTG compared to vehicle GRK$^{-}\beta_1$AR;miR-150 DTG are shown in **C**, and DE lncRNAs in ISO GRK$^{-}\beta_1$AR;miR-150 DTG compared to ISO GRK$^{-}\beta_1$AR TG at 7 days post-treatment are shown in **D.**

Figure S5

mRNA profiling

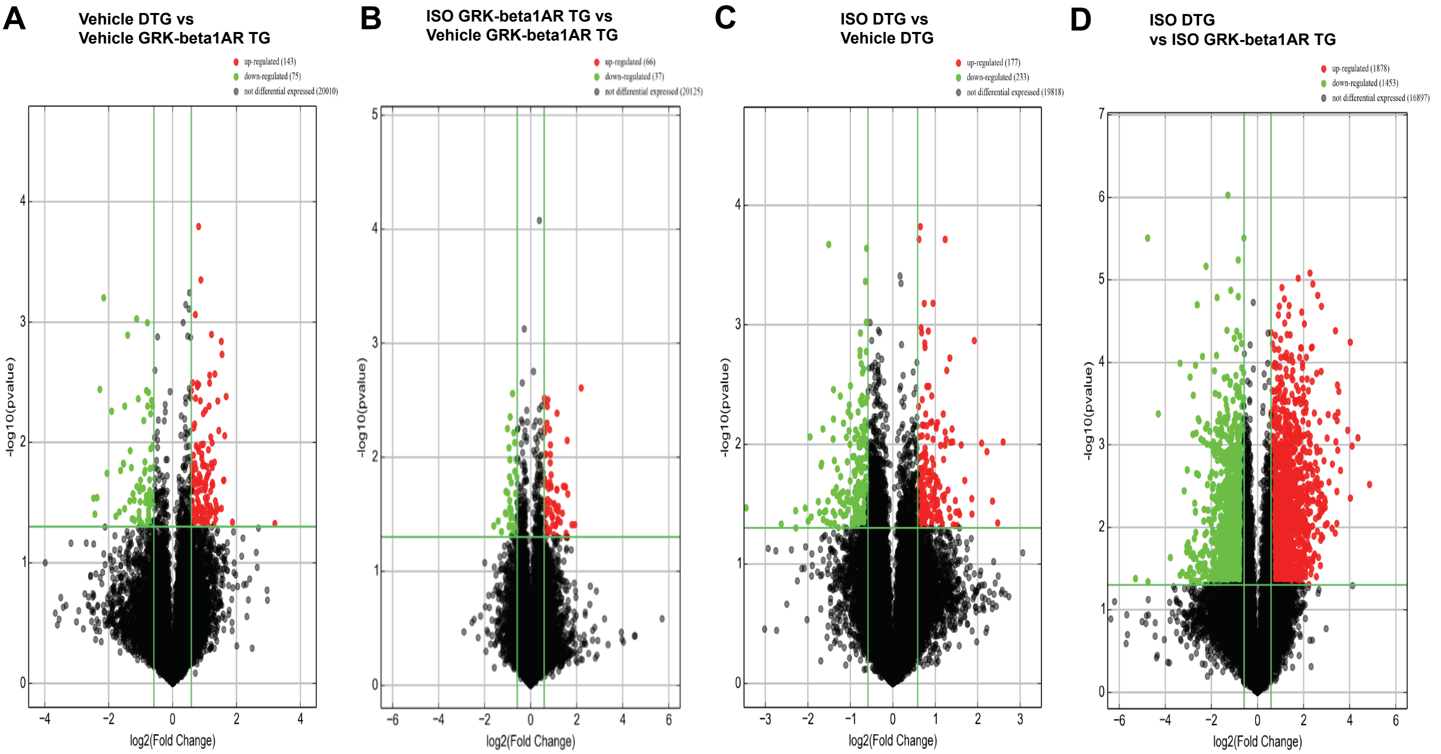

Figure S5. Volcano plot analyses of differentially expressed cardiac genes to discover gene signatures that are regulated by β_1AR/β-arrestin-mediated signaling selectively in cardiomyocytes and control miR-150. A-D, Volcano Plots are made using fold-change values and *P* values that allow visualization of the relationship between fold-change and statistical significance, taking both magnitudes of change and variability into consideration. The vertical lines correspond to 1.5-fold up and down, and the horizontal line represents a *P* value of 0.05. So, the red (up) or green (down) points in the plot represent the differentially expressed (DE) genes with statistical significance. DE genes in vehicle GRK$^{-}\beta_1$AR;miR-150 DTG compared to vehicle GRK$^{-}\beta_1$AR TG controls are shown in **A**, DE genes in ISO GRK$^{-}\beta_1$AR TG compared to vehicle GRK$^{-}\beta_1$AR TG controls are shown in **B**, DE genes in ISO GRK$^{-}\beta_1$AR;miR-150 DTG compared to vehicle GRK$^{-}\beta_1$AR;miR-150 DTG are shown in **C**, and DE genes in ISO GRK$^{-}\beta_1$AR;miR-150 DTG compared to ISO GRK$^{-}\beta_1$AR TG at 7 days post-treatment are shown in **D.**

Figure S6

Upregulated Signaling Pathways

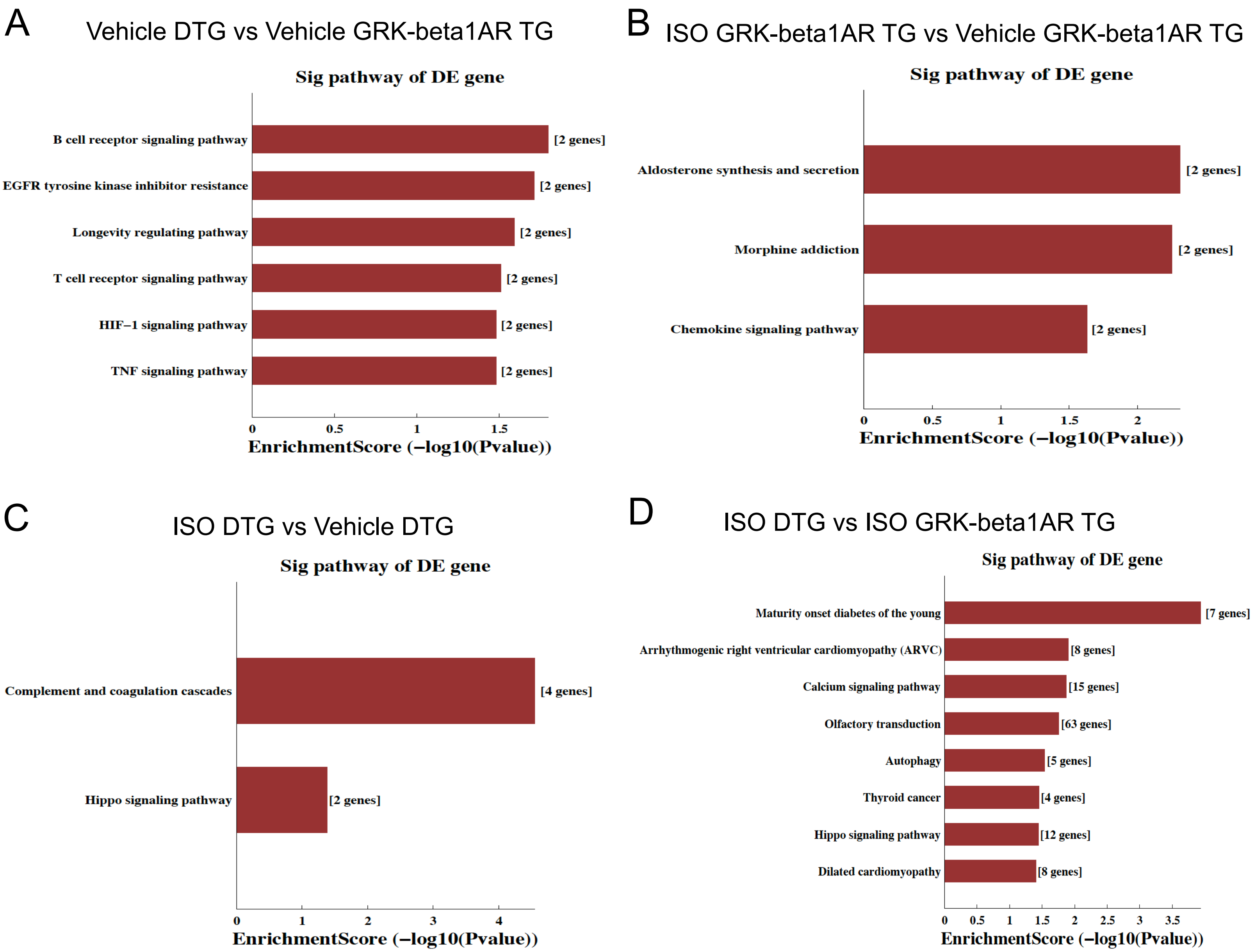

Figure S6. Signaling pathway analyses of differentially upregulated genes. The functional pathway analyses are performed by mapping differentially expressed (DE) genes to Kyoto Encyclopedia of Genes and Genomes (KEGG) pathways. Fisher's exact test is used. The *P* value indicates the significance of the pathway between groups (**A:** vehicle GRK$^{-}\beta_1$AR;miR-150 DTG compared to vehicle GRK$^{-}\beta_1$AR TG controls, **B:** ISO GRK$^{-}\beta_1$AR TG compared to vehicle GRK$^{-}\beta_1$AR TG controls, **C:** ISO GRK$^{-}\beta_1$AR;miR-150 DTG compared to vehicle GRK$^{-}\beta_1$AR;miR-150 DTG, and **D:** ISO GRK$^{-}\beta_1$AR;miR-150 DTG compared to ISO GRK$^{-}\beta_1$AR TG). Enrichment Score is the value of the Pathway ID, which equals to -log10(Pvalue). The bar plots represent the top enrichment score values of significant enrichment pathways. Among DE genes discovered in microarray analyses (Table S5), upregulated genes are only shown.

Figure S7

Downregulated Signaling Pathways

A Vehicle DTG vs Vehicle GRK-beta1AR TG

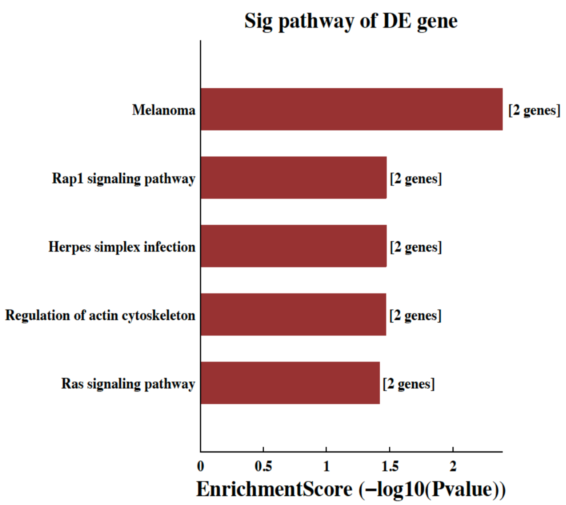

B ISO DTG vs Vehicle DTG

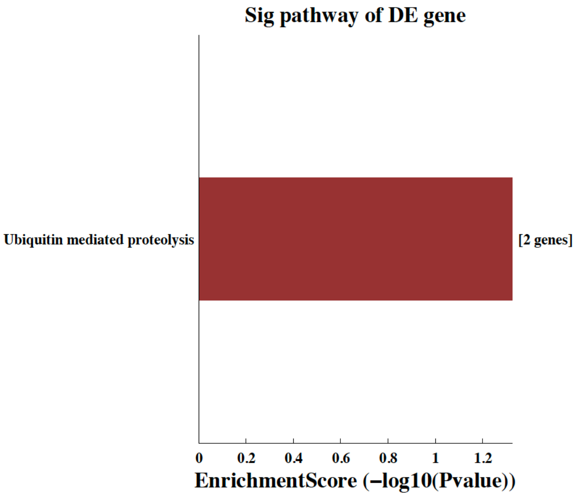

C ISO DTG vs ISO GRK-beta1AR TG

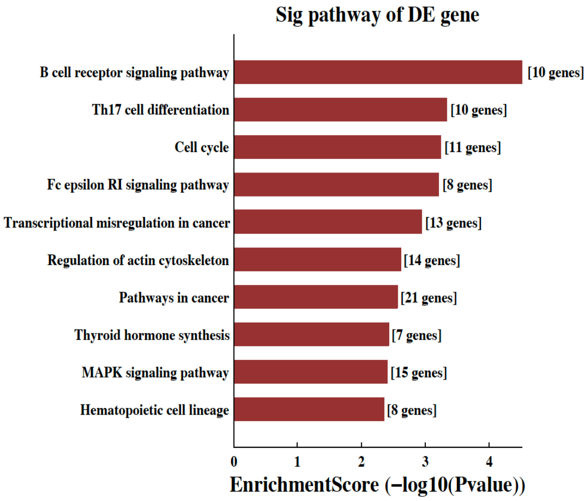

Figure S7. Signaling pathway analyses of differentially downregulated genes. The functional pathway analyses are performed by mapping differentially expressed (DE) genes to Kyoto Encyclopedia of Genes and Genomes (KEGG) pathways. Fisher's exact test is used. The *P* value indicates the significance of the pathway between groups (**A:** vehicle GRK$^{-}\beta_1$AR;miR-150 DTG compared to vehicle GRK$^{-}\beta_1$AR TG controls, **B:** ISO GRK$^{-}\beta_1$AR;miR-150 DTG compared to vehicle GRK$^{-}\beta_1$AR;miR-150 DTG, and **C:** ISO GRK$^{-}\beta_1$AR;miR-150 DTG compared to ISO GRK$^{-}\beta_1$AR TG). Enrichment Score is the value of the Pathway ID, which equals to -log10(Pvalue). The bar plots represent the top enrichment score values of significant enrichment pathways. Among DE genes discovered in microarray analyses (Table S5), downregulated genes are only shown.

Figure S8

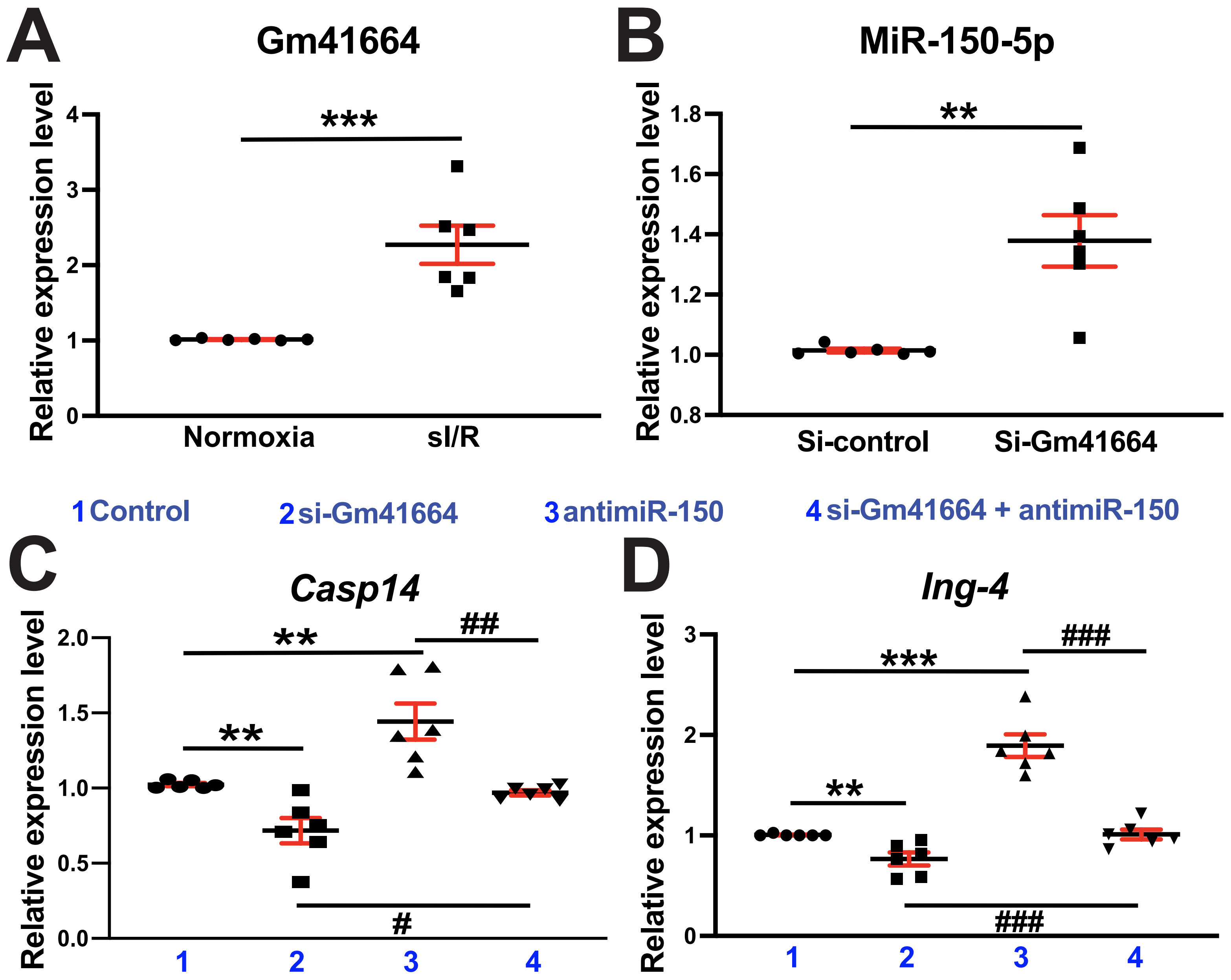

A
Gm41664
Relative expression level
***
Normoxia
sI/R
B
MiR-150-5p
Relative expression level
**
Si-control
Si-Gm41664
1 Control
2 si-Gm41664
3 antimiR-150
4 si-Gm41664 + antimiR-150
C
Casp14
Relative expression level
**
##
**
#
D
Ing-4
Relative expression level
***
###
**
###

Figure S8. Gm41664 represses miR-150 in cardiomyocytes, and miR-150 is necessary for Gm41664-dependent regulation of pro-apoptotic *Casp14* or *Ing4* expression in cardiomyocytes. A, Gm41664 is increased in CMs subjected to *in vitro* simulation of I/R (hypoxia/reoxygenation) [sI/R (H/R)]. HL-1 cells were subjected to sI/R. N=6. *Gm41664* expression compared to *Gapdh* was calculated using $2^{-\Delta\Delta Ct}$, and data are presented as fold induction of *Gm41664* expression levels normalized to normoxia. Unpaired 2-tailed t-test. ****P*<0.01 vs. normoxia. **B**, HL-1 cells were transfected with control scramble siRNA (si-control) or Gm41664 siRNA (si-Gm41664). QRT-PCR for miR-150 was performed. Data were then normalized to *U6 snRNA* and expressed relative to si-control. N=6 per group. Unpaired 2-tailed t-test. ***P*<0.01 vs. si-control. **C-D**, MiR-150 knockdown reverses the decreased pro-apoptotic gene expression mediated by Gm41664 knockdown in CMs. QRT-PCR expression analysis of pro-apoptotic *Casp14* and *Ing-4* in CMs transfected with 4 different groups as indicated. N=6. *Casp14* and *Ing-4* expression compared to *Gapdh* was calculated using $2^{-\Delta\Delta Ct}$, and data are presented as fold induction of *Casp14* and *Ing-4* expression levels normalized to control (si-control or antimiR control). One-way ANOVA with Tukey multiple comparison test. ***P*<0.01 or ****P*<0.001 vs. control. $^{\#}$*P*<0.05, $^{\#\#}$*P*<0.01 or $^{\#\#\#}$*P*<0.001 vs. si-Gm41664 + antimiR-150.

Figure S9

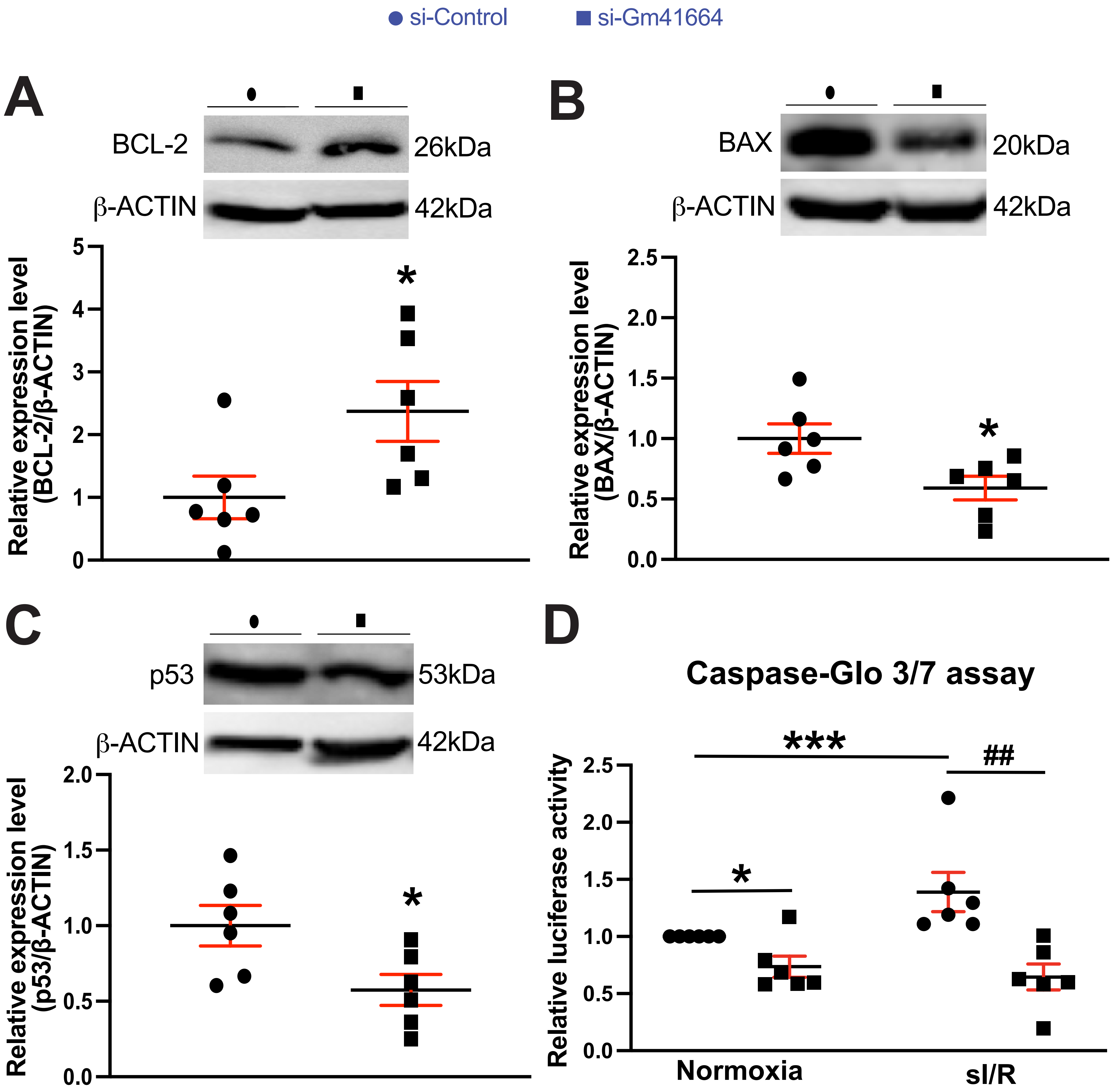
● si-Control
■ si-Gm41664
A
BCL-2
26kDa
β-ACTIN
42kDa
Relative expression level (BCL-2/β-ACTIN)
B
BAX
20kDa
β-ACTIN
42kDa
Relative expression level (BAX/β-ACTIN)
C
p53
53kDa
β-ACTIN
42kDa
Relative expression level (p53/β-ACTIN)
D
Caspase-Glo 3/7 assay
Relative luciferase activity
Normoxia
sI/R
***
##

Figure S9. Gm41664 induces cardiomyocyte apoptosis. A-C, Gm41664 knockdown in CMs increases antiapoptotic BCL-2 and decreases proapoptotic levels of BAX and p53. HL-1 cells were transfected with control scramble siRNA (si-Control) or Gm41664 siRNA (si-Gm41664). Western Blotting analyses of antiapoptotic BCL-2 (**A**) and proapoptotic BAX and p53 (**B-C**) were performed. N=6 per group. Data are presented as fold induction of protein expression normalized to β-ACTIN. Unpaired 2-tailed t-test. $^{*}P<0.05$ vs. si-Control. **D,** Gm41664 knockdown decreases caspase-3/7 activity. HL-1 cells were transfected with si-Control or si-Gm41664 and subjected to *in vitro* simulation of I/R (hypoxia/reoxygenation) [sI/R (H/R)]. Caspase-Glo 3/7 luciferase assay was then performed. Data were expressed relative to si-Control normoxia. N=6 per group. One-way ANOVA with Tukey multiple comparison test. $^{*}P<0.05$ or $^{***}P<0.001$ vs. si-control normoxia. $^{\#\#}P<0.01$ vs. si-control normoxia sI/R.

Figure S10

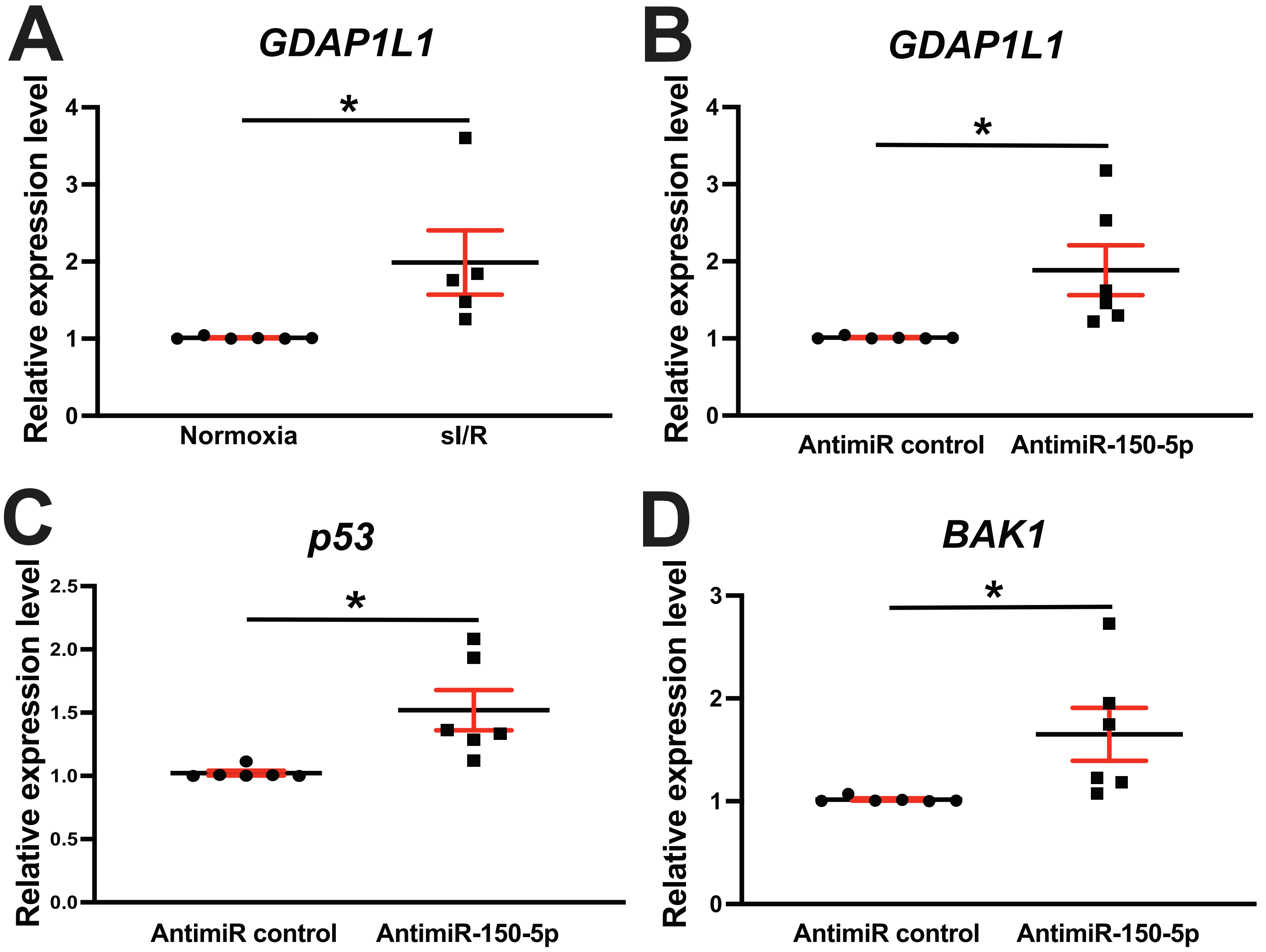
A
GDAP1L1
Relative expression level
0
1
2
3
4
*
Normoxia
sI/R
B
GDAP1L1
Relative expression level
0
1
2
3
4
*
AntimiR control
AntimiR-150-5p
C
p53
Relative expression level
0.0
0.5
1.0
1.5
2.0
2.5
*
AntimiR control
AntimiR-150-5p
D
BAK1
Relative expression level
0
1
2
3
*
AntimiR control
AntimiR-150-5p

Figure S10. *GDAP1L1* is repressed by pro-survival miR-150 in human cardiomyocytes. A, *GDAP1L1* is increased in human cardiomyocytes (HuCMs) subjected to *in vitro* simulation of I/R (hypoxia/reoxygenation) [sI/R (H/R)]. AC16 cells were subjected to sI/R. N=5-6. *GDAP1L1* expression compared to *GAPDH* was calculated using $2^{-\Delta\Delta Ct}$, and data are presented as fold induction of *GDAP1L1* expression levels normalized to normoxia. Unpaired 2-tailed t-test. **P*<0.05 vs. normoxia. **B-D**, AC16 cells were transfected with antimiR control or antimiR-150. QRT-PCR for pro-apoptotic *GDAP1L1* (**B**), *p53* (**C**), and *BAK1* (**D**) was performed. Data were then normalized to *GAPDH* and expressed relative to antimiR control. N=6 per group. Unpaired 2-tailed t-test. **P*<0.05 vs. antimiR control.

Figure S11

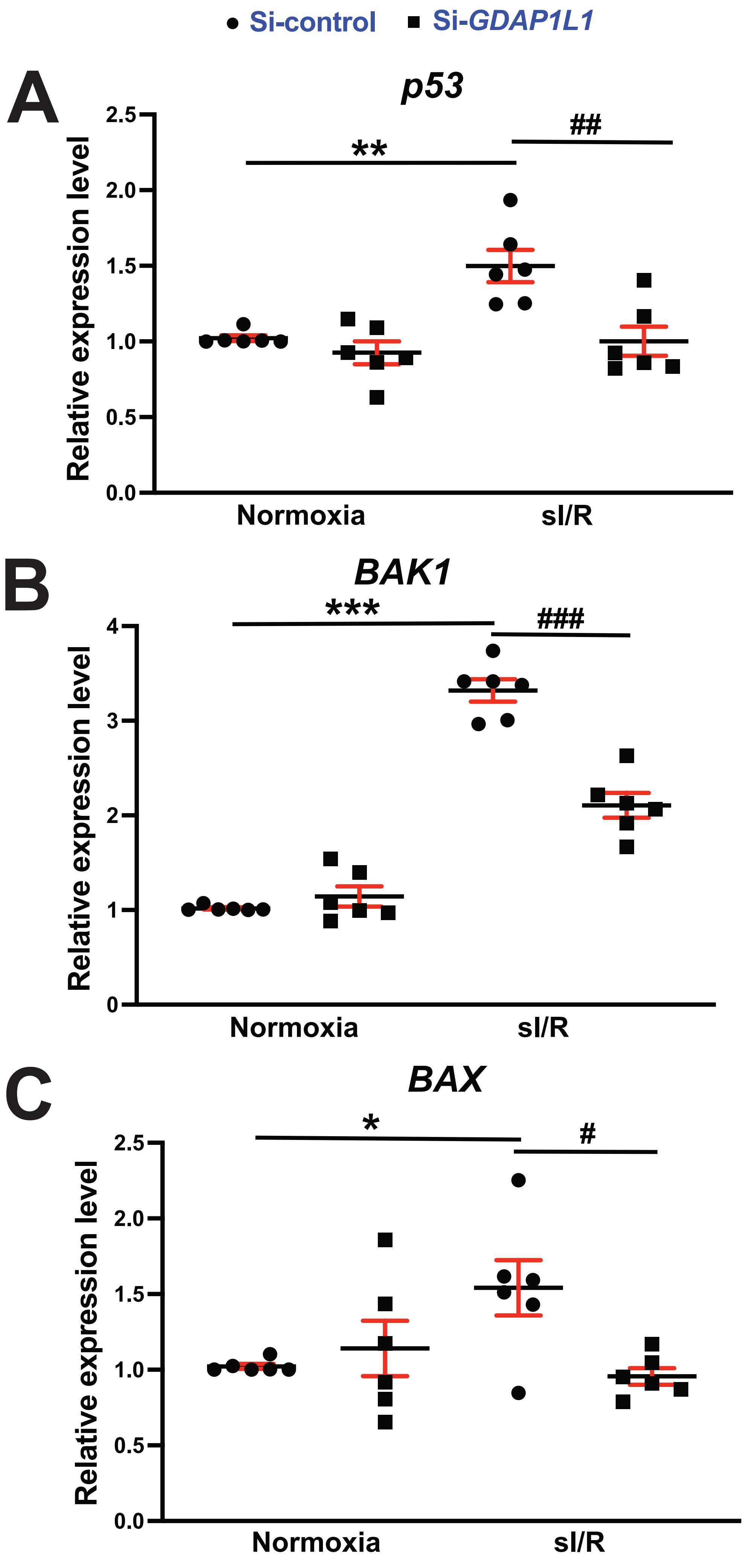

Si-control
Si-GDAP1L1
A
p53
Relative expression level
2.5
2.0
1.5
1.0
0.5
0.0
**
##
Normoxia
sI/R
B
BAK1
Relative expression level
4
3
2
1
0
***
###
Normoxia
sI/R
C
BAX
Relative expression level
2.5
2.0
1.5
1.0
0.5
0.0
*
#
Normoxia
sI/R

Figure S11. *GDAP1L1* activates pro-apoptotic gene expression in human cardiomyocytes. **A-C**, AC-16 cells were transfected with control scramble siRNA (si-control) or *GDAP1L1* siRNA (si-*GDAP1L1*) and were subjected to *in vitro* simulation of I/R (hypoxia/reoxygenation) [sI/R (H/R)]. QRT-PCR expression analyses of pro-apoptotic *p53, BAK1* and *BAX* in human cardiomyocytes (HuCMs) were then conducted. N=6. *p53, BAK1* and *BAX* expression compared to *GAPDH* was calculated using $2^{-\Delta\Delta Ct}$, and data are shown as fold induction of *p53, BAK1* and *BAX* expression levels normalized to control (si-control normoxia). One-way ANOVA with Tukey multiple comparison test. **P*<0.05, ***P*<0.01 or ****P*<0.001 vs. si-control normoxia. #*P*<0.05, ##*P*<0.01 or ###*P*<0.001 vs. si-control sI/R.

Figure S12

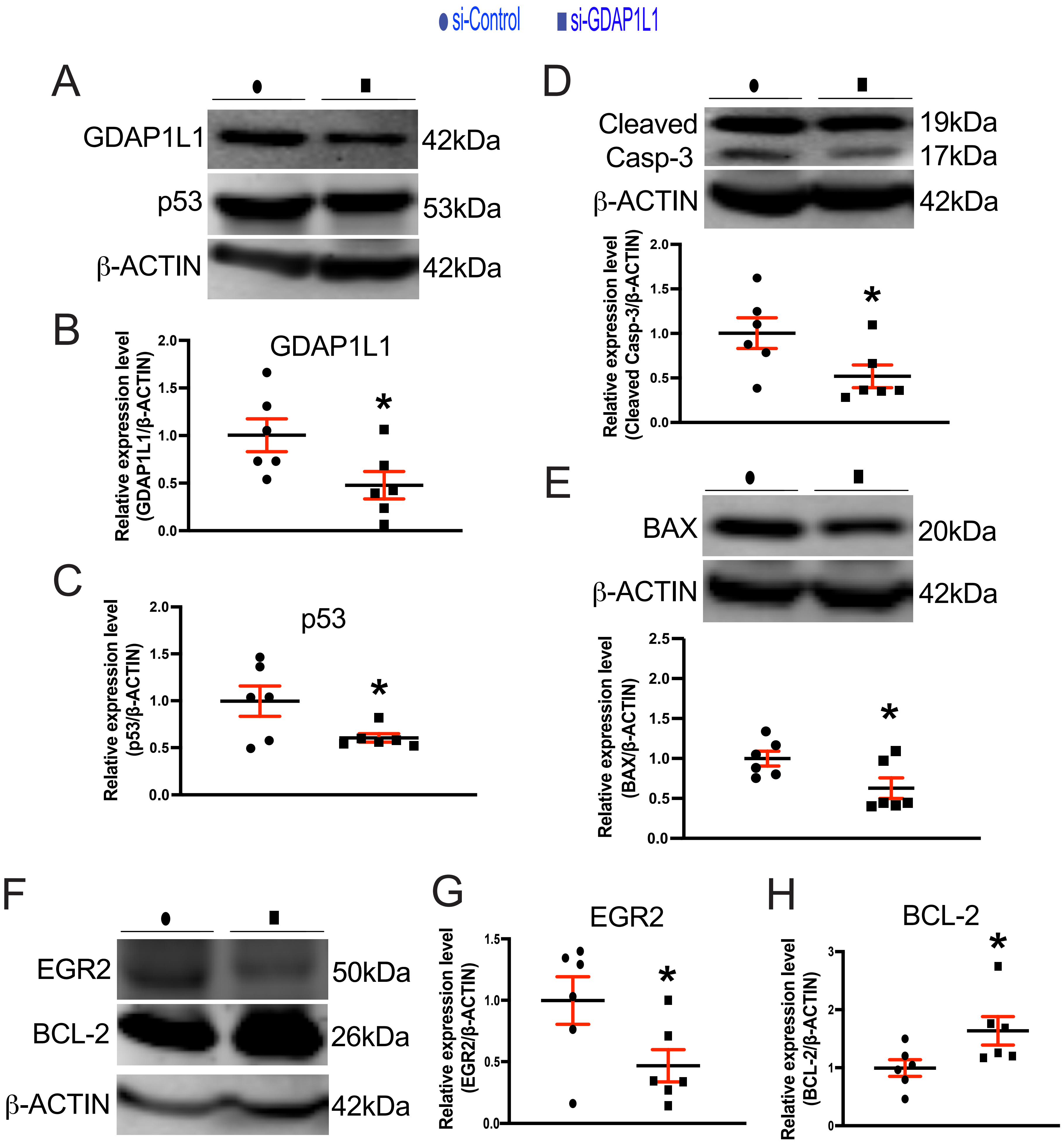
si-Control
si-GDAP1L1
A
GDAP1L1
42kDa
p53
53kDa
β-ACTIN
42kDa
B
GDAP1L1
Relative expression level
(GDAP1L1/β-ACTIN)
C
p53
Relative expression level
(p53/β-ACTIN)
D
Cleaved
Casp-3
19kDa
17kDa
β-ACTIN
42kDa
Relative expression level
(Cleaved Casp-3/β-ACTIN)
E
BAX
20kDa
β-ACTIN
42kDa
Relative expression level
(BAX/β-ACTIN)
F
EGR2
50kDa
BCL-2
26kDa
β-ACTIN
42kDa
G
EGR2
Relative expression level
(EGR2/β-ACTIN)
H
BCL-2
Relative expression level
(BCL-2/β-ACTIN)

Figure S12. *GDAP1L1* knockdown in human cardiomyocytes decreases pro-apoptotic markers, while increasing anti-apoptotic BCL-2. A-H, Human cardiomyocytes (HuCMs) were transfected with control scramble siRNA (si-Control) or *GDAP1L1* siRNA (si-*GDAP1L1*). Western Blotting analysis of GDAP1L1 (**A-B**), pro-apoptotic p53 (**A & C**), CLEAVED CASAPASE-3 (**D**), BAX (**E**), and EGR2 (**F-G**), as well as anti-apoptotic BCL-2 (**F & H**) were performed. *GDAP1L1* knockdown reduces pro-apoptotic protein levels but induces anti-apoptotic BCL-2 in HuCMs. N=6 per group. Data are presented as fold induction of or protein expression normalized to β-ACTIN. Unpaired 2-tailed t-test. *P<0.05 vs. si-Control.

Figure S13

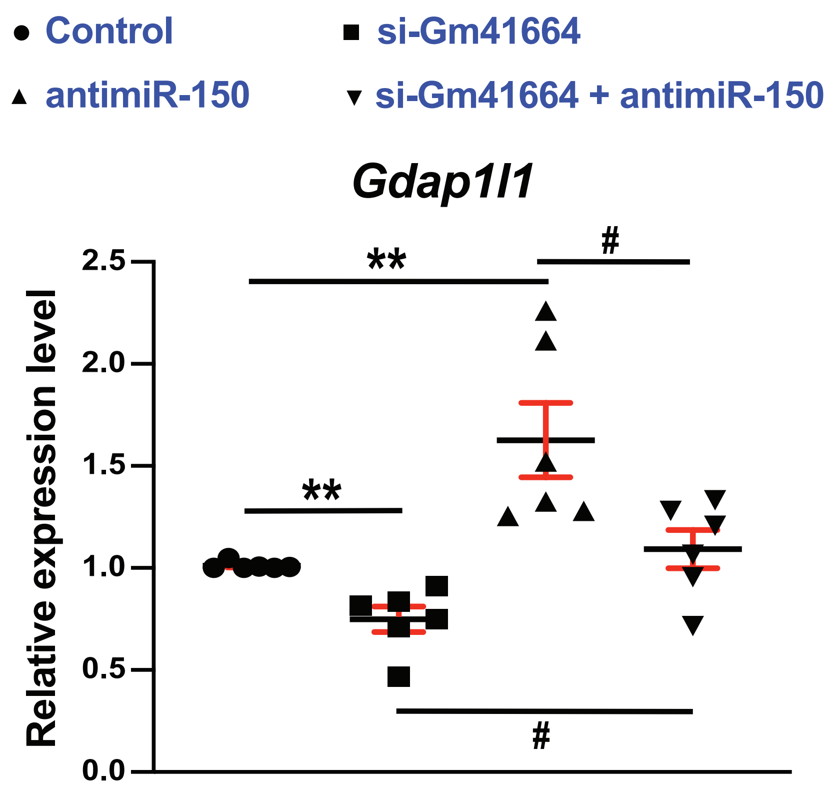
Control
si-Gm41664
antimiR-150
si-Gm41664 + antimiR-150
Gdap1l1
Relative expression level
2.5
2.0
1.5
1.0
0.5
0.0
**
#
**
#

Figure S13. Gm41664 activates *Gdap1l1* in cardiomyocytes in part by repressing miR-150. HL-1 cells were transfected with 4 different groups as indicated. QRT-PCR expression analysis of pro-apoptotic *Gdap1l1* in cardiomyocytes (CMs) were performed. Gm41664 knockdown decreases the expression in *Gdap1l1* in CMs, which is reversed by miR-150 knockdown. N=6. *Gdap1l1* expression compared to *Gapdh* was calculated using $2^{-\Delta\Delta Ct}$. Data are presented as fold induction of *Gdap1l1* expression levels normalized to control (si-control or antimiR control). One-way ANOVA with Tukey multiple comparison test. $^{**}P<0.01$ vs. control. $^{\#}P<0.05$ vs. si-Gm41664 + antimiR-150.

Figure S14

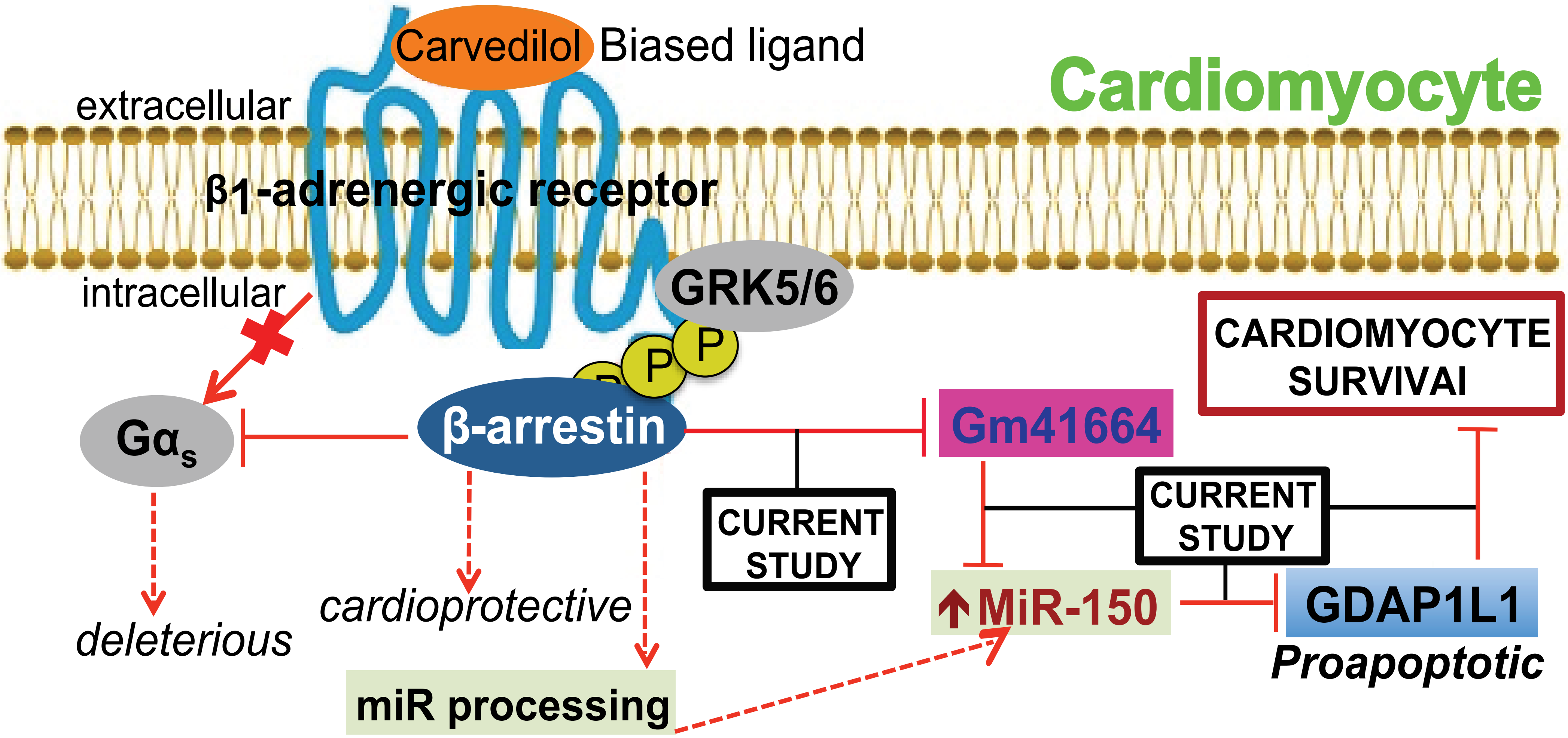
Carvedilol
Biased ligand
Cardiomyocyte
extracellular
β1-adrenergic receptor
intracellular
GRK5/6
P
P
CARDIOMYOCYTE SURVIVAl
Gαs
β-arrestin
Gm41664
CURRENT STUDY
CURRENT STUDY
deleterious
cardioprotective
↑MiR-150
GDAP1L1
Proapoptotic
miR processing

Figure S14. Conceptual framework and schematic illustration summarizing the current study. P: phosphorylated by G protein-coupled receptor kinase (GRK) 5/6. MiR: microRNA. GDAP1L1: Ganglioside induced differentiation associated protein 1 like 1.

Table S1. Echocardiographic parameters in anesthetized GRK⁻β1AR TG and GRK⁻β1AR/miR-150 DTG mice before they were randomly assigned to 4 experimental groups.

Day 0	GRK⁻β1AR TG		GRK⁻β1AR/miR-150 DTG	
	Vehicle (n=18)	Isoproterenol (n=18)	Vehicle (n=18)	Isoproterenol (n=18)
**CO (ml/min)**	23.71 ± 1.33	22.38 ± 1.71	21.89 ± 1.21	22.35 ± 1.42
**EF (%)**	72.64 ± 0.52	72.06 ± 0.51	71.61 ± 0.59	72.36 ± 0.62
**FS (%)**	43.71 ± 0.43	43.55 ± 0.49	43.01 ± 0.39	43.35 ± 0.37
**HR (bpm)**	507 ± 5.71	519 ± 11.05	520 ± 14.83	510 ± 9.57
**SV (µl)**	33.20 ± 1.55	32.42 ± 1.28	32.74 ± 1.05	34.22 ± 3.81
**Volume, diastole (µl)**	50.37 ± 3.43	52.48 ± 2.61	53.83 ± 2.55	51.30 ± 4.29
**Volume, systole (µl)**	14.51 ± 1.33	14.20 ± 0.91	13.85 ± 0.77	12.92 ± 1.04
**LVAW, diastole (mm)**	0.73 ± 0.07	0.79 ± 0.06	0.81 ± 0.06	0.83 ± 0.03
**LVAW, systole (mm)**	1.21 ± 0.05	1.18 ± 0.04	1.22 ± 0.03	1.24 ± 0.07
**LVID, diastole (mm)**	2.53 ± 0.06	2.76 ± 0.07	2.87 ± 0.04	2.65 ± 0.08
**LVID, systole (mm)**	1.53 ± 0.08	1.49 ± 0.04	1.57 ± 0.09	1.51 ± 0.03
**LVPW, diastole (mm)**	0.55 ± 0.04	0.57 ± 0.02	0.58 ± 0.06	0.54 ± 0.05
**LVPW, systole (mm)**	0.73 ± 0.03	0.78 ± 0.08	0.75 ± 0.05	0.79 ± 0.07

Abbreviations: CO = cardiac output, EF = ejection fraction, FS = fractional shortening, HR = heart rate, SV = stroke volume, LVAW = left ventricular anterior wall thickness, LVID = left ventricular interior diameter, LVPW = left ventricular posterior wall thickness. All values are expressed as mean ± SEM.

Table S2. Echocardiographic and morphometric parameters in anesthetized GRK$^-$β1AR TG and GRK$^-$β1AR/miR-150 DTG mice infused with vehicle or isoproterenol for 7 days.

**Day 7**	**GRK$^-$β1AR TG**		**GRK$^-$β1AR/miR-150 DTG**	
**Echocardiography**	**Vehicle (n=18)**	**Isoproterenol (n=18)**	**Vehicle (n=18)**	**Isoproterenol (n=18)**
**CO (ml/min)**	22.38 ± 1.71	15.25 ± 1.87**	23.44 ± 1.14	20.44 ± 1.35#
**EF (%)**	72.62 ± 0.52	56.46 ± 1.08***	71.96 ± 0.71	60.62 ± 0.79***##
**FS (%)**	42.72 ± 0.37	33.57 ± 0.53***	41.85 ± 0.72	36.11 ± 0.56***##
**HR (bpm)**	530 ± 10.37	513 ± 2.72	515 ± 10.41	508 ± 4.88
**SV (µl)**	33.58 ± 1.89	25.72 ± 2.04**	32.11 ± 2.49	30.33 ± 0.83#
**Volume, diastole (µl)**	51.39 ± 3.25	78.84 ± 4.64***	50.55 ± 4.27	59.44 ± 2.39###
**Volume, systole (µl)**	14.59 ± 0.76	38.93 ± 4.88***	14.05 ± 1.02	21.38 ± 0.73***##
**LVAW, diastole (mm)**	0.78 ± 0.05	1.21 ± 0.03***	0.74 ± 0.02	0.88 ± 0.03***###
**LVAW, systole (mm)**	1.27 ± 0.03	1.93 ± 0.07***	1.23 ± 0.04	1.57 ± 0.08***##
**LVID, diastole (mm)**	2.47 ± 0.05	3.27 ± 0.09***	2.54 ± 0.04	2.82 ± 0.06***###
**LVID, systole (mm)**	1.58 ± 0.03	2.33 ± 0.04***	1.55 ± 0.07	1.89 ± 0.03***###
**LVPW, diastole (mm)**	0.52 ± 0.02	0.87 ± 0.06***	0.55 ± 0.03	0.63 ± 0.04##
**LVPW, systole (mm)**	0.71 ± 0.04	1.53 ± 0.03***	0.79 ± 0.08	1.17 ± 0.09**###
**Morphometric data**	**Vehicle (n=12)**	**Isoproterenol (n=12)**	**Vehicle (n=12)**	**Isoproterenol (n=12)**
HW/BW (mg/g)	4.77 ± 0.21	6.96 ± 0.16***	4.95 ± 0.19	5.80 ± 0.27*###
LVW/BW (mg/g)	3.80 ± 0.18	5.88 ± 0.19***	3.44 ± 0.16	4.29 ± 0.26**###

Abbreviations: CO = cardiac output, EF = ejection fraction, FS = fractional shortening, HR = heart rate, SV = stroke volume, LVAW = left ventricular anterior wall thickness, LVID = left ventricular interior diameter, LVPW = left ventricular posterior wall thickness. All values are expressed as mean ± SEM. $^{*}P<0.05$, $^{**}P<0.01$, or $^{***}P<0.001$ vs. vehicle within same group. $^{\#}P<0.05$, $^{\#\#}P<0.01$, or $^{\#\#\#}P<0.001$ vs. GRK$^-$β1AR TG Isoproterenol. Only parameters, which were statistically significant between groups, are highlighted with red fonts.
