# Supplementary material for: MiR-150 blunts cardiac dysfunction in mice with cardiomyocyte loss of β1-adrenergic receptor/β-arrestin signaling and controls a unique transcriptome
Source: Cell Death Discov. 2022 Dec 30;8:504. doi: 10.1038/s41420-022-01295-9 (PMC9803679; doi:10.1038/s41420-022-01295-9)

Figure S9A

Bcl2 26 kDa

Antidody #3498Cell Signaling

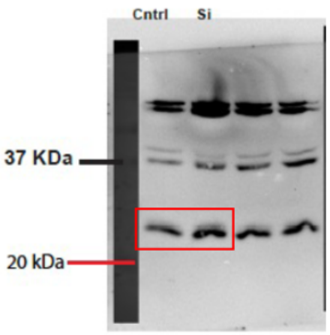

b-actin 42 kDa

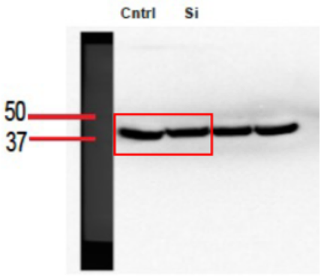

Figure S9B

Bax 20 kDa

Antidody #2772 Cell Signaling

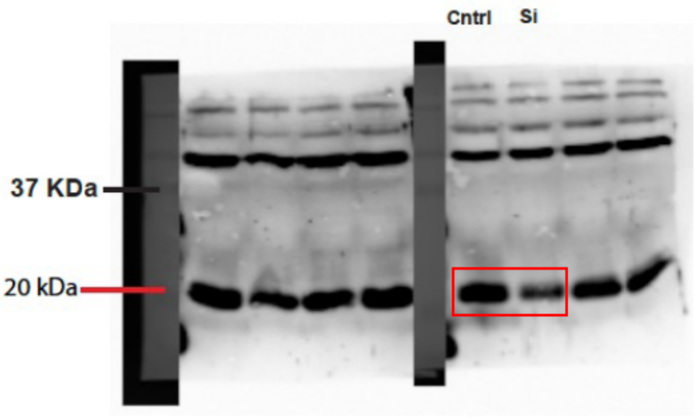

b-actin 42 kDa

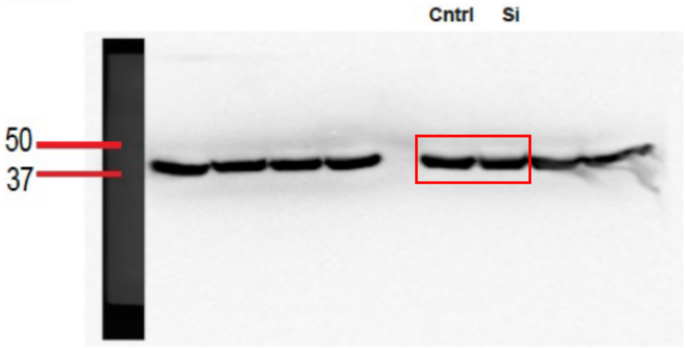

Figure S9C

P53 53 kDa

Antidody #sc-126 Santa cruz biotec

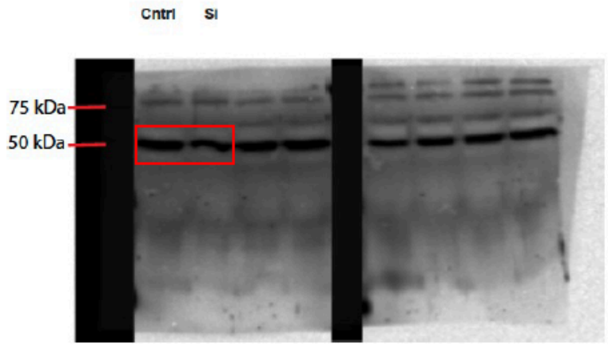

b-actin 42 kDa

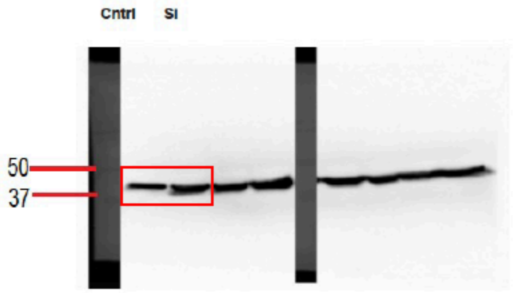

Figure S12A-C

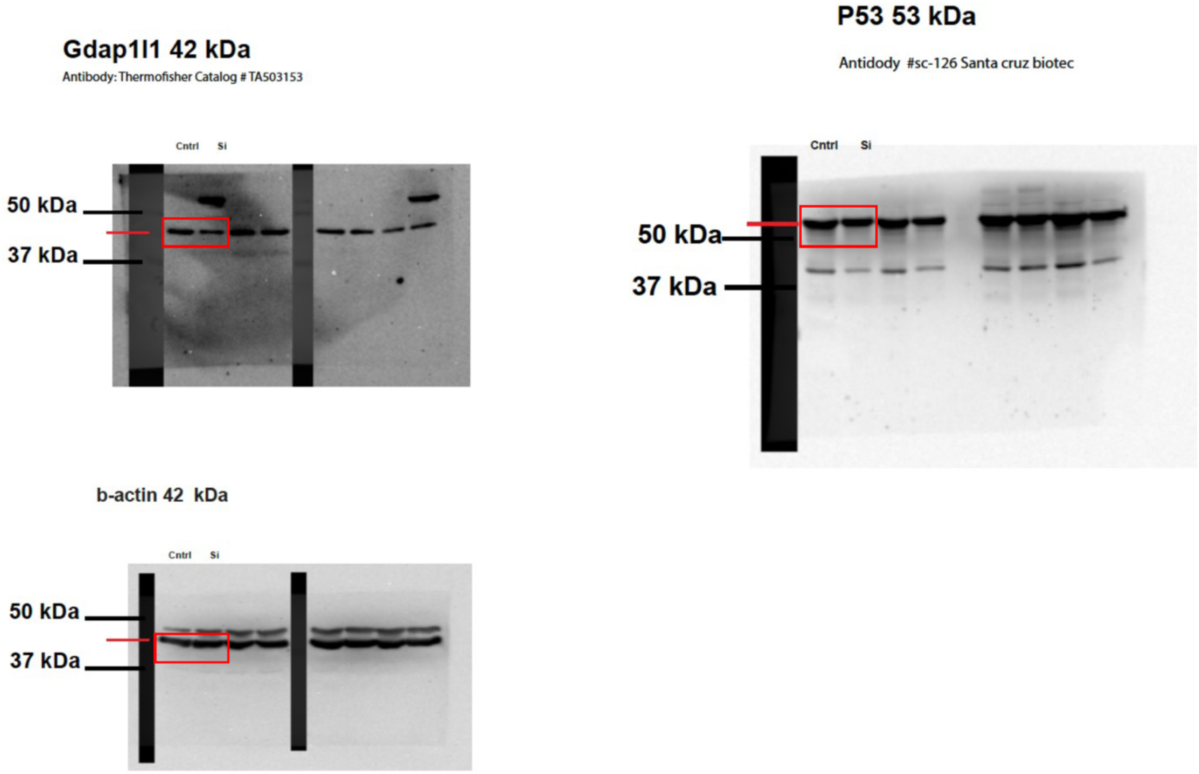

Figure S12D

Cleaved Casp-3 17, 19 kDa

Antibody: Cell Signaling Cat: Antibody #9661

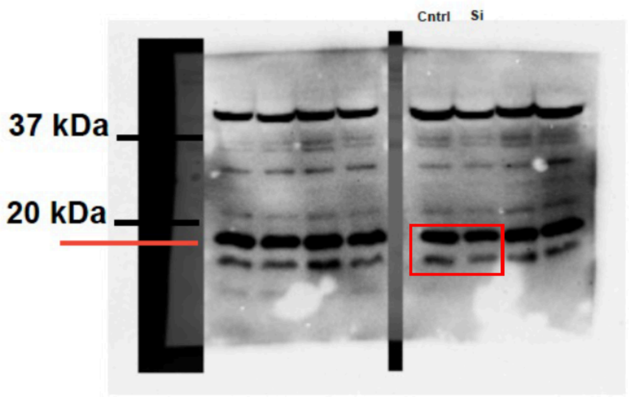

b-actin 42 kDa

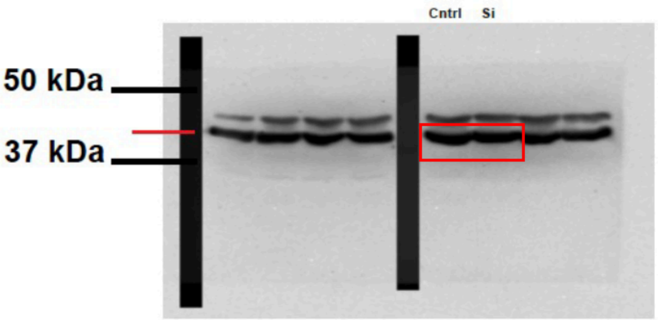

Figure S12E

Bax 20 kDa

Antidody #2772 Cell Signaling

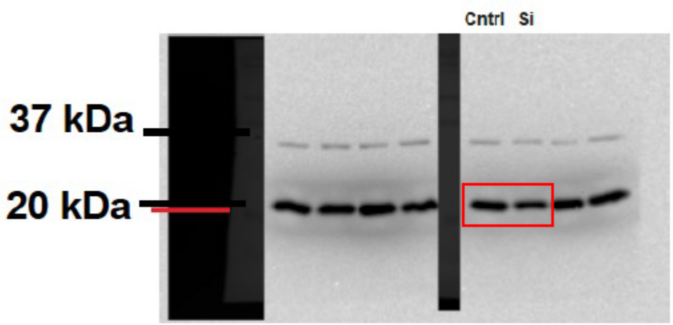

b-actin 42 kDa

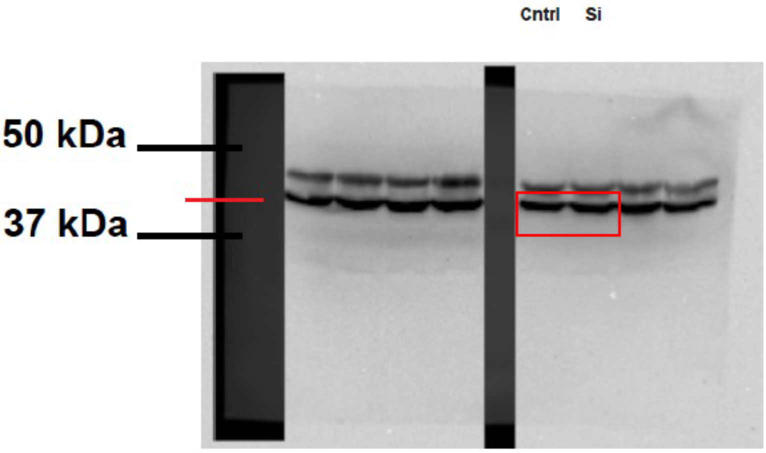

Figure S12F-H

EGR2 50 kDa

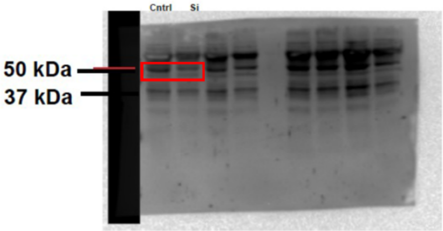

b-actin 42 kDa

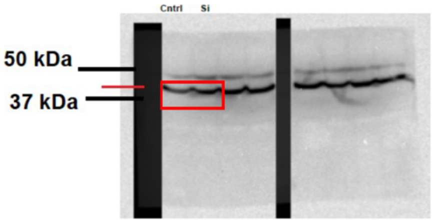

Bcl 2 26 kDa

Antibody: Cell Signaling Bcl-2 (D17C4) Rabbit mAb #3498

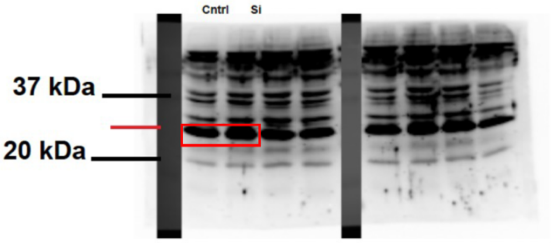

Supplement: Supplementary file 5 — Original Western Blot Images [file 41420_2022_1295_MOESM5_ESM.pdf]
